# Association of COVID-19 With Achieving Time-to-Surgery Benchmarks in Patients With Musculoskeletal Trauma

**DOI:** 10.1001/jamahealthforum.2021.3460

**Published:** 2021-10-29

**Authors:** Ida Leah Gitajn, Paul M. Werth, Sheila Sprague, Nathan O’Hara, Gregory Della Rocca, Robert Zura, Meir Marmor, Christopher M. Domes, Lauren C. Hill, Christine Churchill, Christine Townsend, Chi Van, Natalie Hogan, Cara Girardi, Gerard P. Slobogean

**Affiliations:** 1 Dartmouth-Hitchcock Medical Center, Lebanon, New Hampshire; 2 McMaster University, Hamilton, Ontario, Canada; 3 University of Maryland School of Medicine, Baltimore; 4 University of Missouri, Columbia; 5 Louisiana State University Medical Center, New Orleans; 6 University of California, San Francisco; 7 University of Wisconsin–Madison; 8 IU Health Methodist Hospital, Indianapolis, Indiana; 9 Carolinas Medical Center, Atrium Health Musculoskeletal Institute, Charlotte, North Carolina; 10 Prisma Health, Upstate, Greenville, South Carolina; 11 University of Florida, Gainesville; 12 Vanderbilt Medical Center, Nashville, Tennessee

## Abstract

**Importance:**

In response to the COVID-19 pandemic, many hospital systems were forced to reduce operating room capacity and reallocate resources. The outcomes of these policies on the care of injured patients and the maintenance of emergency services have not been adequately reported.

**Objective:**

To evaluate whether the COVID-19 pandemic was associated with delays in urgent fracture surgery beyond national time-to-surgery benchmarks.

**Design, Setting, and Participants:**

This retrospective cohort study used data collected in the Program of Randomized Trials to Evaluate Preoperative Antiseptic Skin Solutions in Orthopaedic Trauma among at 20 sites throughout the US and Canada and included patients who sustained open fractures or closed femur or hip fractures.

**Exposure:**

COVID-19–era operating room restrictions were compared with pre–COVID-19 data.

**Main Outcomes and Measures:**

Surgery within 24 hours after injury.

**Results:**

A total of 3589 patients (mean [SD] age, 55 [25.4] years; 1913 [53.3%] male) were included in this study, 2175 pre–COVID-19 and 1414 during COVID-19. A total of 54 patients (3.1%) in the open fracture cohort and 407 patients (21.8%) in the closed hip/femur fracture cohort did not meet 24-hour time-to-surgery benchmarks. We were unable to detect any association between time to operating room and COVID-19 era in either open fracture (odds ratio [OR], 1.40; 95% CI, 0.77-2.55; *P* = .28) or closed femur/hip fracture (OR, 1.01; 95% CI, 0.74-1.37; *P* = .97) cohorts. In the closed femur/hip fracture cohort, there was no association between time to operating room and regional COVID-19 prevalence (OR, 1.07; 95% CI, 0.70-1.64; *P* = .76).

**Conclusions and Relevance:**

In this cohort study, there was no association between meeting time-to-surgery benchmarks in either open fracture or closed femur/hip fracture during the COVID-19 pandemic compared with before the pandemic. This is counter to concerns that the unprecedented challenges associated with managing the COVID-19 pandemic would be associated with clinically significant delays in acute management of urgent surgical cases and suggests that many hospital systems within the US were able to effectively implement policies consistent with time-to-surgery standards for orthopedic trauma in the context of COVID-19–related resource constraints.

## Key Points

**Question:**

Were resource constraints due to the COVID-19 pandemic associated with a delay in urgent fracture surgery beyond national time-to-surgery benchmarks?

**Findings:**

In this cohort pre-post study that included 3589 patients, there was no association between time to surgery and COVID-19 in either open fracture or closed femur/hip fracture cohorts.

**Meaning:**

Despite concerns that the unprecedented challenges associated with the COVID-19 pandemic would delay acute management of urgent surgery, many hospital systems within the US were able to implement strategies in keeping with time-to-surgery standards for orthopedic trauma.

## Teaser

This cohort study evaluates whether the COVID-19 pandemic is associated with delays in urgent fracture surgery beyond national time-to-surgery benchmarks.

## Introduction

Since December 2019, the novel SARS-CoV-2 virus has led to more than 123 million worldwide infections and has claimed the lives of more than 2 million people.^1,2^ In response to the COVID-19 pandemic, hospital systems were forced to reduce operating room capacity and reallocate resources. The outcomes of these policies on a hospital’s ability to care for patients with COVID-19 and maintain emergency services have not been adequately reported. The outcomes of these unprecedented policy decisions on the care of acutely injured patients are equally unclear.

The purpose of this study was to evaluate whether the COVID-19 pandemic and subsequent COVID-19–related policies were associated with a delay in urgent fracture surgery beyond national time-to-surgery benchmarks. A 24-hour time from injury to fracture surgery benchmark is recommended for the treatment of open fractures, hip fractures, and femoral shaft fractures to prevent severe infections and mortality. We hypothesized that COVID-19 policies would be associated with a lower proportion of acutely injured patients receiving care within this national guideline and that this association would vary depending on the rate of regional COVID-19 cases.

## Methods

### Study Design and Procedures

This cohort study was a secondary analysis of data collected in the Program of Randomized Trials to Evaluate Preoperative Antiseptic Skin Solutions in Orthopaedic Trauma (PREP-IT) program, which comprises 2 parallel cluster randomized crossover trials: A Pragmatic Randomized Trial Evaluating Preoperative Aqueous Antiseptic Skin Solutions in Open Fractures (Aqueous-PREP)^3^ (NCT03385304) and A Pragmatic Randomized Trial Evaluating Preoperative Alcohol Skin Solutions in Fractured Extremities (PREPARE)^3^ (NCT03523962). Enrollment took place in 20 sites across the US and Canada. PREP-IT trial participants were required to be adults (aged 18 years or older), presenting with an open fracture of an extremity requiring surgery or a closed lower extremity or pelvis fracture requiring surgery. This study has a blanket approval to use the data collected for secondary analysis from all participating institutional review boards, including the Methods Centre REB. In addition, when data sharing agreements are prepared, the Methods Centre REB is required to sign off. This study followed the Strengthening the Reporting of Observational Studies in Epidemiology (STROBE) reporting guideline for cohort studies.

The PREP-IT trials compare iodophor vs chlorhexidine-based antiseptic skin preparation solutions. Clinical sites were randomized to determine which study solution to begin using, and each site crossed over to the alternative study solution every 2 months. The primary benefit of the cluster randomized crossover trial design is that all patients treated at the recruiting hospitals will receive the predetermined study intervention prior to patient enrollment. This efficiency maximizes recruitment feasibility because study consent does not need to occur prior to the patient’s urgent surgery and it minimizes selection bias to improve internal and external study validity. Because of this, study participation has no effect on time to surgery. Study participant race and ethnicity were based on self-report and verified in the electronic health record.

For the current retrospective cohort study, all patients included in the PREP-IT program at sites who had begun enrolling pre–COVID-19 who sustained an open fracture, femur fracture (Orthopaedic Trauma Association classification 32A, 32B, 32C, 33A, 33B, 33C), or hip fracture (Orthopaedic Trauma Association classification 31A, 31B, 31C) were eligible for inclusion. The outcome investigated was time to surgery bifurcated into surgery within 24 hours of hospital admittance or greater than 24 hours from hospital admittance. No patients were excluded from the analysis. Patient cohorts examined were included from multiple institutions as part of the PREP-IT program. Institutions were eligible for this secondary analysis if they reported treating eligible patients both prior to and during the COVID-19 pandemic. The primary focus of this study is to determine whether hospital policy changes related to COVID-19 were associated with time to surgery. March 13, 2020, was selected as the threshold when the COVID-19 policy period started because this is when a federally mandated declaration of national emergency was announced. Patients admitted to 1 of the PREPARE participating sites between the dates of March 13, 2019, and March 12, 2020, were dummy coded as the control group representing the pre–COVID-19 period, and patients admitted between the dates of March 13, 2020, and January 31, 2021, were dummy coded as the intervention group representing the COVID-19 pandemic period subject to emergency policy changes.

Additional analyses were conducted to determine whether regional rates of COVID-19 infection were associated with the same binary time-to-surgery outcome. To determine regional rates of infection, US county population estimates^4^ and Canadian census data collected from specified hospital site regions as well as number of daily positive COVID-19 viral test results for each county or region in which a participating institution sits were populated from March 13, 2020, through January 2021 using the continuously collected data set compiled by the New York Times^5^ and for each Canadian region. The ratio of positive viral test results to estimated population size for each county was determined for each day. Change in day-over-day rates were used as the focal independent variable for this analysis in the closed fracture cohorts.

### Statistical Analysis

#### Data Transformations

All analyses were conducted in the R environment, version 4.0.3 (R Foundation for Statistical Computing).^6^ Univariate tests were conducted across relevant patient characteristics stratified by period (ie, pre–COVID-19 vs COVID-19) for both open and closed fracture cohorts. Continuous variables were reported as means and SDs, and comparisons were performed with use of independent-sample *t* tests. Categorical variables were reported as frequencies and percentages. Missingness was assessed for all statistical models. Significant missingness was discovered for the injury severity score variable for both open and closed fracture cohorts. Multiple imputation by chained equations was used to remediate missingness in this injury severity score, accounting for the hierarchical structure of the data.

#### Statistical Modeling

Binary logistic mixed effects modeling with random intercepts was used to investigate all research questions. Considering that patient data were derived from multiple institutions, hospital site was entered into each model as a random effect. Due to minimal sizes in certain categories of the American Society of Anesthesiologists (ASA) class variable, categories were collapsed for the closed cohort model. Age was centered on hospital site mean. For the open fracture cohort, injury severity score underwent a log transformation. For the closed fracture cohort, fracture location and an interaction term between fracture location (femoral shaft vs proximal femur) and COVID-19 period were included as independent variables. The interaction term was further probed to understand the association of fracture location and COVID-19 period with the time-to-surgery outcome.

For the analysis investigating regional COVID-19 infection rates for the closed fracture cohort, age, ASA class, injury severity score, fracture location, and COVID-19 regional rate were included as control variables (same as the prior regression model). Because of some sites having so few cases that did not meet 24-hour time-to-surgery benchmarks, we had insufficient power and were unable to investigate regional COVID-19 infection rate for the open fracture cohort.

Considering the use of multiple imputation procedures, Rubin’s rules for pooled estimates are reported for all analyses.^7^ A post hoc power analysis was performed with regard to the open fracture cohort because it had the least statistical power of the 2 cohorts. Assuming a type I error of .05, the current sample size achieved power of .83. The following inputs were used at this power: observed sample size, intercept odds ratio (OR) of 0.01, probability of 0.035 for missing the 24-hour surgical window pre–COVID-19, type I error of .05, and shared *R*^2^ of 0.1 between the COVID-19 period variable and other independent variables in the model. For *P* values, a 2-tailed *t* test was performed and level of significance was set at .05.

## Results

A total of 3598 patients from 20 medical centers across the US and Canada were included. Patient demographics and injury characteristics are summarized in Table 1 for the open fracture cohort and Table 2 for the closed fracture cohort. Mean (SD) age in the open fracture cohort was 45.4 (18.2) years with 1100 (63.4%) men. Mean (SD) age in the closed fracture cohort was 66.7 (20.3) years with 813 (43.6%) men. Patient characteristics stratified by COVID-19 period for both open and closed fracture cohorts are presented. Significant differences in age (mean [SD], 46.4 [18.5] years pre–COVID-19 vs 43.7 [17.8] years during COVID-19; *P* = .003) and fracture contamination (pre–COVID-19 vs during COVID-19 there was none/minimal, 686 [61.2%] vs 398 [65.4%]; surface contamination, 340 [30.4%] vs 147 [24.1%]; contaminant embedded, 94 [8.3%] vs 64 [10.5%]; *P* = .01) were observed in the open fracture cohort (Table 1). Significant differences in age (mean [SD], 66.8 [20.3] years pre–COVID-19 vs 61.7 [21.5] years during COVID-19; *P* < .001), sex (429 [40.5%] male pre–COVID-19 vs 384 [47.7%] during COVID-19; *P* = .002), race and ethnicity, ASA class, and injury mechanism were observed in the closed fracture cohort (Table 2). A total of 54 patients (3.1%) in the open fracture cohort and 407 patients (21.8%) in the closed hip/femur fracture cohort did not meet 24-hour time-to-surgery benchmarks. There was variability around ORs for meeting 24-hour time-to-surgery benchmarks across hospital site (eFigures 1 and 2 and eTable 1 in Supplement 1) (range, 73.9% to 100% in the open fracture cohort and 48.4% to 100% in the closed fracture cohort).

**Table 1.** Patient Characteristics Stratified by Onset of COVID-19 Pandemic in the US for the Open Fracture Cohort^a^

Characteristic	No. (%)		*P* value
	Pre–COVID-19	COVID-19	
Total, No.	1126	609	NA
Age, mean (SD)	46.4 (18.5)	43.7 (17.8)	.003
Sex			
Female	431 (38.3)	204 (33.5)	.11
Male	695 (61.7)	405 (66.5)	
BMI, mean (SD)	29.2 (7.3)	29.0 (7.1)	.52
BMI category			
Underweight (<18.5)	13 (1.2)	10 (1.6)	.94
Normal weight (18.5-24.9)	332 (29.5)	178 (29.2)	
Overweight (25.0-29.9)	362 (32.1)	201 (33.0)	
Class I obesity (30.0-34.9)	224 (19.9)	113 (18.6)	
Class II obesity (35.0-39.9)	98 (8.7)	52 (8.5)	
Class III obesity (≥40.0)	97 (8.6)	55 (9.0)	
Race and ethnicity			
American Indian/Alaska Native	13 (1.2)	7 (1.1)	.34
Asian	13 (1.2)	6 (1.0)	
Black/African American	207 (18.4)	137 (22.5)	
White	747 (66.3)	379 (62.2)	
Other^b^	129 (11.5)	73 (11.8)	
Unknown	17 (1.5)	7 (1.1)	
ASA class^c^			
I	111 (9.9)	55 (9.0)	.87
II	485 (43.1)	274 (45.0)	
III	410 (36.4)	221 (36.3)	
IV	109 (9.7)	55 (9.0)	
V	11 (1.0)	4 (0.7)	
Gustilo-Anderson classification			
I	256 (22.7)	158 (25.9)	.11
II	368 (32.7)	178 (29.2)	
IIIA	438 (38.9)	229 (37.6)	
IIIB	52 (4.6)	30 (4.9)	
IIIC	12 (1.1)	14 (2.3)	
Extremity = upper^d^	288 (25.6)	149 (24.5)	.65
Fracture contamination			
None or minimal	689 (61.2)	398 (65.4)	.01
Surface contamination	343 (30.5)	147 (24.1)	
Contaminant embedded	94 (8.3)	64 (10.5)	
Injury mechanism			
Fall	300 (26.6)	147 (24.1)	.19
Motor vehicle accident	637 (56.6)	340 (55.8)	
Other	189 (16.8)	122 (20.0)	
Injury Severity Score, mean (SD)	1.70 (0.98)	1.66 (1.03)	.51
Time to surgery >24 h	30 (2.7)	24 (3.9)	.19

Abbreviations: BMI, body mass index (calculated as weight in kilograms divided by height in meters squared); NA, not applicable.

^a^ Univariate tests across time period include independent samples *t* tests for continuous variables and χ^2^ tests for categorical variables.

^b^ Other includes American Indian or Alaska Native, Native Hawaiian, Pacific Islander, or multiple races.

^c^ American Society of Anesthesiologists Physical Status Classification System.

^d^ Lower extremity.

**Table 2.** Patient Characteristics Stratified by Onset of COVID-19 Pandemic in the US for the Closed Fracture Cohort^a^

Characteristic	No. (%)		*P* value
	Pre–COVID-19	COVID-19	
Total, No.	1058	805	NA
Age, mean (SD)	66.8 (20.3)	61.7 (21.5)	<.001
Sex			
Female	629 (59.5)	421 (52.3)	.002
Male	429 (40.5)	384 (47.7)	
BMI, mean (SD)	27.2 (7.2)	27.5 (7.4)	.34
BMI category			
Underweight (<18.5)	51 (4.8)	36 (4.5)	.70
Normal weight (18.5-24.9)	412 (38.9)	301 (37.5)	
Overweight (25.0-29.9)	318 (30.1)	234 (29.2)	
Class I obesity (30.0-34.9)	150 (14.2)	133 (16.6)	
Class II obesity (35.0-39.9)	66 (6.2)	45 (5.6)	
Class III obesity (≥40.0)	61 (5.8)	53 (6.6)	
Race and ethnicity			
American Indian/Alaska Native	2 (0.2)	3 (0.4)	.02
Asian	4 (0.4)	9 (1.1)	
Black/African American	119 (11.2)	130 (16.1)	
White	708 (66.9)	505 (62.7)	
Other^b^	216 (20.4)	151 (18.6)	
Unknown	9 (0.9)	7 (0.9)	
ASA class^c^			
I	40 (3.8)	33 (4.1)	<.001
II	281 (26.6)	290 (36.0)	
III	608 (57.5)	390 (48.4)	
IV	128 (12.1)	89 (11.1)	
V	1 (0.1)	3 (0.4)	
Injury mechanism			
Fall	812 (76.7)	520 (64.6)	<.001
Motor vehicle accident	191 (18.1)	234 (29.1)	
Other	55 (5.2)	51 (6.3)	
Fracture location = femur^d^	407 (38.5)	327 (40.6)	.37
Injury Severity Score, mean (SD)	9.75 (4.96)	9.68 (5.78)	.84
Time to surgery >24 h	244 (23.1)	163 (20.2)	.16

Abbreviations: BMI, body mass index (calculated as weight in kilograms divided by height in meters squared); NA, not applicable.

^a^ Univariate tests across time period include independent samples *t* tests for continuous variables and χ^2^ tests for categorical variables.

^b^ Other includes American Indian or Alaska Native, Native Hawaiian, Pacific Islander, or multiple races.

^c^ American Society of Anesthesiologists Physical Status Classification System.

^d^ Hip fracture.

In the open fracture cohort, 30 patients (2.7%) did not meet the less-than-24-hour time-to–operating room benchmarks pre–COVID-19 and 24 patients (3.90%) did not meet less-than-24-hour time-to–operating room benchmarks during the COVID-19 era (*P* = .19). After controlling for hypothesis-driven variables, there was no independent association between admission during COVID-19 and delay to the operating room beyond 24 hours in the open fracture cohort (OR, 1.40; 95% CI, 0.77-2.55; *P* = .28) (Table 3). Older age (OR, 1.02; 95% CI, 1.01-1.04; *P* = .02) and Injury Severity Score (OR, 2.53; 95% CI, 1.53-4.18; *P* < .001) were independently associated with lower odds of meeting time-to–operating room benchmarks (Table 3).

**Table 3.** Logistic Mixed Effects Model With Multiple Imputation Examining Time to Surgery for Open Fracture

Variable	DV: surgery within 24 h [Y/N]^a^		
	Odds ratio (95% CI)	*P* value	RIV
Intercept	0.01 (0.00-0.02)	<.001	0.13
Age^b^	1.02 (1.01-1.04)	.02	0.06
Fracture contamination^c^			
Surface	0.67 (0.34-1.33)	.25	0.02
Embedded	0.27 (0.06-1.21)	.09	<0.01
Injury Severity Score^d^	2.53 (1.53-4.18)	<.001	0.55
COVID-19 era: yes	1.40 (0.77-2.55)	.28	0.02
Random effects			
ICC	0.51	NA	NA
No. of hospital sites	24	NA	NA
Observations	1735	NA	NA

Abbreviations: DV, dependent variable; ICC, intraclass correlation coefficient; NA, not applicable; RIV, relative increases in variance.

^a^ Referent: yes.

^b^ Centered on hospital site mean.

^c^ Referent: none or minimal.

^d^ Log_2_ transformation.

Among femur and hip fractures, 244 patients (23.1%) did not meet less-than-24-hour time-to–operating room benchmarks pre–COVID-19 and 163 patients (20.2%) did not meet less-than-24-hour time-to–operating room benchmarks during the COVID-19 era (*P* = .16). There was no significant difference between meeting time-to–operating room benchmarks between hip and femur fractures (81.4% vs 80.4%; *P* = .27). After controlling for hypothesis-driven variables, there was no independent association between admission during COVID-19 and delay to the operating room beyond 24 hours in the femur/hip fracture cohort (OR, 1.01; 95% CI, 0.74-1.37; *P* = .97) (Table 4). Older age (OR, 1.01; 95% CI, 1.01-1.02; *P* = .009) and ASA class IV or V (OR, 2.99; 95% CI, 1.98-4.53; *P* < .001) were associated with greater odds of not meeting time-to–operating room benchmarks. Fracture location was not a significantly associated with time to surgery (OR, 1.15; 95% CI, 0.83-1.61; *P* = .40) (Table 4, eFigure 3 in Supplement 1). The interaction variable between COVID-19 era and fracture location was also not significantly associated with time to surgery (OR, 0.88; 95% CI, 0.54-1.45; *P* = .62). Among femur and hip fractures, when evaluating the association between meeting less-than-24-hour time-to–operating room benchmarks and regional COVID-19 case rate, there was still no association between regional COVID-19 rate and meeting time-to–operating room benchmarks (OR, 1.07; 95% CI, 0.70-1.64; *P* = .76) (Table 5).

**Table 4.** Logistic Mixed Effects Model With Multiple Imputation Examining Time to Surgery for Closed Fracture

Variable	DV: surgery within 24 h [Y/N]^a^		
	Odds ratio (95% CI)	*P* value	RIV
Intercept	0.13 (0.08-0.22)	<.001	0.02
Age^b^	1.01 (1.01-1.02)	.009	0.01
ASA class^c^			
III	1.30 (0.96-1.74)	.09	<0.01
IV or V	2.99 (1.98-4.53)	<.001	<0.01
Injury Severity Score	1.01 (0.98-1.04)	.65	0.08
COVID-19 era: yes	1.01 (0.74-1.37)	.97	<0.01
Fracture location: femur	1.15 (0.83-1.61)	.40	<0.01
COVID-19 era: yes × fracture location: femur^d^	0.88 (0.54-1.45)	.62	<0.01
Random effects			
ICC	0.32	NA	NA
No. of hospital sites	19	NA	NA
Observations	1869	NA	NA

Abbreviations: DV, dependent variable; ICC, intraclass correlation coefficient; NA, not applicable; RIV, relative increases in variance.

^a^ Referent: yes.

^b^ Centered on hospital site mean.

^c^ Referent: class I or II.

^d^ Moderated effect or interaction term between these 2 variables.

**Table 5.** Logistic Mixed Effects Model With Multiple Imputation Examining Time to Surgery for Closed Fracture Accounting for Regional COVID-19 Case Rates

Variable	DV: surgery within 24 h [Y/N]^a^		
	Odds ratio (95% CI)	*P* value	RIV
Intercept	0.10 (0.04-0.23)	<.001	0.15
Timing variable, mo	1.10 (0.97-1.24)	.12	0.21
Age^b^	1.01 (0.99-1.02)	.30	0.04
ASA class^c^			
III	1.38 (0.88-2.17)	.16	0.01
IV or V	1.81 (0.88-3.72)	.11	0.08
Injury severity score	0.99 (0.93-1.06)	.78	1.15
Fracture location: femur	1.00 (0.99-1.02)	.90	0.13
COVID-19 regional rate	1.07 (0.70-1.64)	.76	0.02
COVID-19 regional rate × fracture location: femur^d^	1.00 (0.98-1.03)	.76	0.28
Random effects			
ICC	0.41	NA	NA
No. of hospital sites	19	NA	NA
Observations	807	NA	NA

Abbreviations: DV, dependent variable; ICC, intraclass correlation coefficient; NA, not applicable; RIV, relative increases in variance.

^a^ Referent: yes.

^b^ Centered on hospital site mean.

^c^ Referent: class I or II.

^d^ Moderated effect or interaction term between these 2 variables.

When the association between race and delay beyond benchmarks were assessed, there was no association between race and meeting 24-hour time-to-surgery benchmarks in the open fracture cohort. However, non-White race was associated with increased odds of not meeting 24-hour time-to-surgery benchmarks in the hip/femur fracture cohort (OR, 2.93; 95% CI, 2.04-4.27; *P* < .001) (eTables 2 and 3 in Supplement 1).

There was a reduction in the number of patients screened who sustained study-eligible traumatic injuries and the number of patients enrolled in March and April, which rebounded in May and June, reflecting both the association of COVID-19 with research infrastructure and patient enrollment as well as the volume of trauma at these centers (eFigure 4 in Supplement 1).

## Discussion

In this cohort study, there was no association between meeting time-to-surgery benchmarks in either open fracture or closed femur/hip fracture during the COVID-19 pandemic compared with before the pandemic. The COVID-19 pandemic has created unprecedented challenges to our global and national health care systems, stressing our resources and shifting protocols around clinical management strategies. As a result of increasingly limited resources, many hospital systems were forced to reduce operating room capacity and reallocate resources, which raised concern around delays in acute management of patients with traumatic injury. However, contrary to the hypothesis, in this cohort study of patients enrolled at 1 of 20 sites into the PREP-IT program, the COVID-19 pandemic was not associated with the ability of hospital systems to meet 24-hour time-to-surgery benchmarks for open fractures, hip fractures, and femur fractures during the COVID-19 pandemic. It is particularly striking that there was a rate of only 3% for delay to the operating room beyond 24 hours in the open fracture group. In the femur/hip fracture cohort, there was a 22% rate of delay beyond 24 hours.

There are several potential reasons for these results, which likely vary across institutions. Overall, it is reassuring that hospitals included in this study were able to effectively manage operating room resources to ensure timely care of acutely injured patients. Owing to the large scale of this study and its retrospective nature, it was not possible to report which specific components of trauma workflow may have contributed to the preservation of standard time-to–operating room protocols. It is possible that a reduction in trauma volumes contributed to this, as many fields saw an acute reduction in the number of patients presenting for acute issues, such as stroke,^8,9^ myocardial infarction,^10,11^ and emergency surgery.^12^ In the UK and Scotland, trauma admissions were reported to be down during the lockdown compared with prior years.^13,14^ This was thought to be associated with collateral effects of social distancing and avoidance of health care institutions due to fear of contracting the virus.^15^ There was a reduction in the number of patients who were screened and who were enrolled into the PREP-IT cohort after COVID-19. However, this may be, in part, due to hospital-based protocols around restricting research activities, which varied in intensity and length of time across institutions. Comprehensive national database analyses are needed to assess the association of COVID-19 with trauma volumes and effectively assess changes in distribution of trauma during COVID-19. Another likely reason why COVID-19 protocols seemingly did not interfere with the ability to maintain time-to-surgery benchmarks is that institutions were able to offset the increase in resource and time needs associated with COVID-19 protocols, such as donning and doffing personal protective equipment and decontamination of critical health care resources, through the reduction in elective surgical procedures, freeing up operating room and staffing resources.

Additional studies are needed to address whether there was a change in patient outcomes after traumatic injury. Acute traumatic injury and fracture may be protected from some of the issues with delayed presentation seen in conditions such as appendicitis,^16^ emergency general surgery,^17^ myocardial infarction,^10,11^ and stroke^9^ because it is more difficult to minimize or ignore symptoms associated with an unstable fracture or high-energy traumatic injury. The early studies evaluating associations of COVID-19 with patient outcomes in orthopedic trauma have demonstrated mixed results. In Scotland, MacDonald et al^14^ reported that mortality among orthopedic trauma patients was higher in March through May of 2020 than in March through May of 2019 (5.0% in 2020 vs 2.8% in 2019 and 1.8% in 2018). However, Donovan et al^18^ reported no difference in complications or mortality among orthopedic trauma patients during COVID-19 in 1 trauma center in the UK. They did, however, note longer mean operating room times for orthopedic trauma patients, which was thought to be due to new personal protective equipment requirements.^18^ Increased mortality may be attributed to presence of COVID-19 in trauma patients or in-hospital transmission. However, targeted research is needed to explore this on a wider scale.

The 3% rate of delay beyond 24 hours in the open fracture group was reassuring and was actually so uncommon that it limited our analytic power. This study did identify a relatively high rate of greater-than-24-hour time to operating room of 22% in the hip/femur fracture group, which was similar in both the hip and femur fracture subgroups. Delayed time to operating room in the open fracture cohort had an independent association with less severe fracture contamination and higher Injury Severity Score. These associations are not surprising, as surgeons likely feel increasingly motivated to ensure early time to operating room in more highly contaminated injuries and necessary surgery is most frequently delayed by increasing injury severity in remote regions, resulting in critical systemic condition. In the hip/femur fracture cohort, delayed time to operating room was independently associated with higher ASA class, which reflects delays due to medical complexity.

### Limitations

There are several limitations associated with this study. Because failure to meet time-to-surgery benchmarks was rare in the open fracture group (only 3%), there was a concern about the potential for underpower. However, a post hoc power analysis was performed and demonstrated that even in the open fracture cohort (with the least statistical power), this sample size achieved power of .83, which is above the generally accepted rate. Furthermore, in both cohorts, a higher proportion of patients met time-to-surgery benchmarks after the start of COVID-19 (although not statistically significant), which substantiates the conclusion that COVID-19–related resource reallocation does not appear to have had a detrimental association with meeting time-to-surgery benchmarks. Furthermore, the low rate of delayed time to surgery throughout the entire study time is reassuring, broadly speaking. While this study did not capture data on the presence of COVID-19 infection in acutely injured patients or at specific institutions enrolling patients, the rate of infection is likely related to the regional rates, which was factored into the second multivariate model in closed fractures. Because of the large scale of this study and its retrospective nature, it was not possible to report hospital-specific policies. Additional research is needed to evaluate the outcome of specific policies on ability to meet benchmarks. Although there are no published or accepted values for a clinically important difference, the less than 3% differences between the groups in both cohorts are unlikely to represent a clinically relevant difference. Because the rate of not meeting the less-than-24-hour time-to-surgery benchmarks was low in open fracture patients, there was not enough statistical power to evaluate the association between regional COVID-19 case rate and time to operating room. Although the reasons underlying the less-than-24-hour time-to–operating room standards in closed femur and closed hip fractures differ, these were evaluated in a single closed fracture cohort. A single model approach was used for both hip and femur fractures because the same set of independent variables was relevant for inclusion in the regression model. This has the benefit of a larger sample size and thus more statistical power, which helps to alleviate issues associated with imbalance in outcome (the majority of patients received surgery within 24 hours). An interaction term was included, which evaluates the independent association of fracture type (femur vs hip) with COVID-19 variables, and there was no independent association of this interaction term with the time-to-surgery metric. There was a high degree of missingness in Injury Severity Scores, which is an important factor in considering time to surgery. However, the risk of bias associated with this was mitigated using multiple imputation procedures. Because the PREPARE trial was not designed to assess the outcomes of the COVID-19 pandemic, the groups are not evenly distributed and there are differences between the groups. However, multivariate regression was used to control for these differences, as is indicated for retrospective or observational research.

There were several strengths associated with this study. The high-quality prospectively collected data represent a substantial strength. Furthermore, 2 multivariate models were created in an effort to answer this question, assessing any change in time-to–operating room benchmarks from pre–COVID-19 to after the federally mandated declaration of a national emergency was announced and also based on regional COVID-19 rates derived from the Centers for Disease Control and Prevention. This analysis included a large sample size adding to the power and inclusion of 20 sites, which expands generalizability. The multicenter nature of this study allowed us to evaluate the broad associations of the COVID-19 pandemic on a national scale, as opposed to a single center.

## Conclusions

Although the COVID-19 pandemic has created unprecedented challenge to health care systems far and wide, this cohort study suggests that hospital systems throughout the US and Canada were able to effectively mitigate these challenges such that 24-hour time-to-surgery benchmarks were unchanged from before the pandemic to after the pandemic.

## Notes

Supplement 1. **eTable 1.** Patients Numbers by Site Stratified by Onset of COVID-19 for Both Open and Closed Fracture Cohorts **eTable 2.** Evaluation of Race in Open Fracture Cohort **eTable 3.** Evaluation of Race in Closed Fracture Cohort **eFigure 1.** Random Effects of Hospital Sites for Open Fracture Cohort **eFigure 2.** Random Effects of Hospital Sites for Closed Fracture Cohort **eFigure 3.** Hip and Femur Fracture Interaction Plot **eFigure 4.** Patient Enrollment Plot

Supplement 2. PREP-IT Investigators Nonauthor Collaborators
