# Supplementary material for: Association of COVID-19 With Achieving Time-to-Surgery Benchmarks in Patients With Musculoskeletal Trauma
Source: JAMA Health Forum. Author manuscript; Available in PMC 2022 Jan 18. (PMC8727030; doi:10.1001/jamahealthforum.2021.3460)
Supplement: Supplement 1 — eTable 1. Patients Numbers by Site Stratified by Onset of COVID-19 for Both Open and Closed Fracture Cohorts eTable 2. Evaluation of Race in Open Fracture Cohort eTable 3. Evaluation of Race in Closed Fracture Cohort eFigure 1. Random Effects of Hospital Sites for Open Fracture Cohort eFigure 2. Random Effects of Hospital Sites for Closed Fracture Cohort eFigure 3. Hip and Femur Fracture Interaction Plot eFigure 4. Patient Enrollment Plot [file NIHMS1771809-supplement-Supplement_1.pdf]

Supplemental Online Content

Gitajn IL, Werth PM, Sprague S, et al; for the PREP-IT Investigators. Association of COVID-19 with achieving time-to-surgery benchmarks in patients with musculoskeletal trauma. *JAMA Health Forum*. 2021;2(10):e213460. doi:10.1001/jamahealthforum.2021.3460

eTable 1. Patients Numbers by Site Stratified by Onset of COVID-19 for Both Open and Closed Fracture Cohorts

eTable 2. Evaluation of Race in Open Fracture Cohort

eTable 3. Evaluation of Race in Closed Fracture Cohort

eFigure 1. Random Effects of Hospital Sites for Open Fracture Cohort

eFigure 2. Random Effects of Hospital Sites for Closed Fracture Cohort

eFigure 3. Hip and Femur Fracture Interaction Plot

eFigure 4. Patient Enrollment Plot

This supplemental material has been provided by the authors to give readers additional information about their work.

© 2021 Gitajn IL et al. *JAMA Health Forum.*

**eTable 1.** ***Patients Numbers by Site Stratified by Onset of COVID-19 for Both Open and Closed Fracture Cohorts***				
	Open Fracture		Closed Fracture	
	**Pre-COVID**	**COVID**	**Pre-COVID**	**COVID**
**Total n**	1126	609	1062	807
**Site** (%)				
1	24 (2.1)	4 (0.7)	-	-
2	15 (1.3)	8 (1.3)	57 (5.4)	22 (2.7)
3	23 (2.0)	21 (3.4)	53 (5.0)	34 (4.2)
4	23 (2.0)	16 (2.6)	63 (5.9)	31 (3.8)
5	79 (7.0)	11 (1.8)	26 (2.4)	27 (3.3)
6	91 (8.1)	44 (7.2)	17 (1.6)	29 (3.6)
7	40 (3.6)	28 (4.6)	84 (7.9)	61 (7.6)
8	101 (9.0)	46 (7.6)	33 (3.1)	42 (5.2)
9	19 (1.7)	3 (0.5)	51 (4.8)	13 (1.6)
10	40 (3.6)	25 (4.1)	67 (6.3)	29 (3.6)
11	-	-	45 (4.2)	59 (7.3)
12	8 (0.7)	19 (3.1)	36 (3.4)	35 (4.3)
13	26 (2.3)	15 (2.5)	32 (3.0)	38 (4.7)
14	148 (13.1)	111 (18.2)	85 (8.0)	85 (10.5)
15	28 (2.5)	21 (3.4)	192 (18.1)	119 (14.7)
16	64 (5.7)	16 (2.6)	1 (0.1)	35 (4.3)
17	2 (0.2)	12 (2.0)	8 (0.8)	20 (2.5)
18	18 (1.6)	14 (2.3)	93 (8.8)	62 (7.7)
19	23 (2.0)	5 (0.8)	-	-
20	98 (8.7)	20 (3.3)	-	-
21	29 (2.6)	17 (2.8)	70 (6.6)	34 (4.2)
22	71 (6.3)	79 (13.0)	-	-
23	89 (7.9)	47 (7.7)	49 (4.6)	32 (4.0)
24	23 (2.0)	26 (4.3)	-	-
25	44 (3.9)	1 (0.2)	-	-

© 2021 Gitajn IL et al. *JAMA Health Forum.*

**eTable 2. Evaluation of Race in Open Fracture Cohort**<br>*Univariate Binary Logistic Regression Model*			
	**DV[1]: Surgery within 24 hours [Y/N]**		
Variable	*Odds Ratios*	*CI*	*p*
(Intercept)	0.04	0.02 – 0.07	**<0.001**
Race[2] [Other[3]]	0.36	0.10 – 1.02	0.075
Race [White]	0.78	0.42 – 1.51	0.436
Observations	1725		
R^2 Tjur	0.002		
Note: [1]Referent: Yes. [2]Referent: Black/African American. [3]Other categories: Asian, American Indian or Alaska Native, Native Hawaiian or Other Pacific Islander, Other.			

© 2021 Gitajn IL et al. *JAMA Health Forum.*

**eTable 3. Evaluation of Race in Closed Fracture Cohort**<br>*Univariate Binary Logistic Regression Model*			
	**DV[1]: Surgery within 24 hours [Y/N]**		
Predictors	*Odds Ratios*	*CI*	*p*
(Intercept)	0.25	0.18 – 0.33	**<0.001**
Race: *Other*	2.93	2.04 – 4.27	**<0.001**
Race: *White*	0.77	0.55 – 1. 01	0.138
Observations	1869		
R^2 Tjur	0.064		
Note: [1]Referent: Black or African American.			

© 2021 Gitajn IL et al. *JAMA Health Forum.*

eFigure 1. Random Effects of Hospital Sites for Open Fracture Cohort

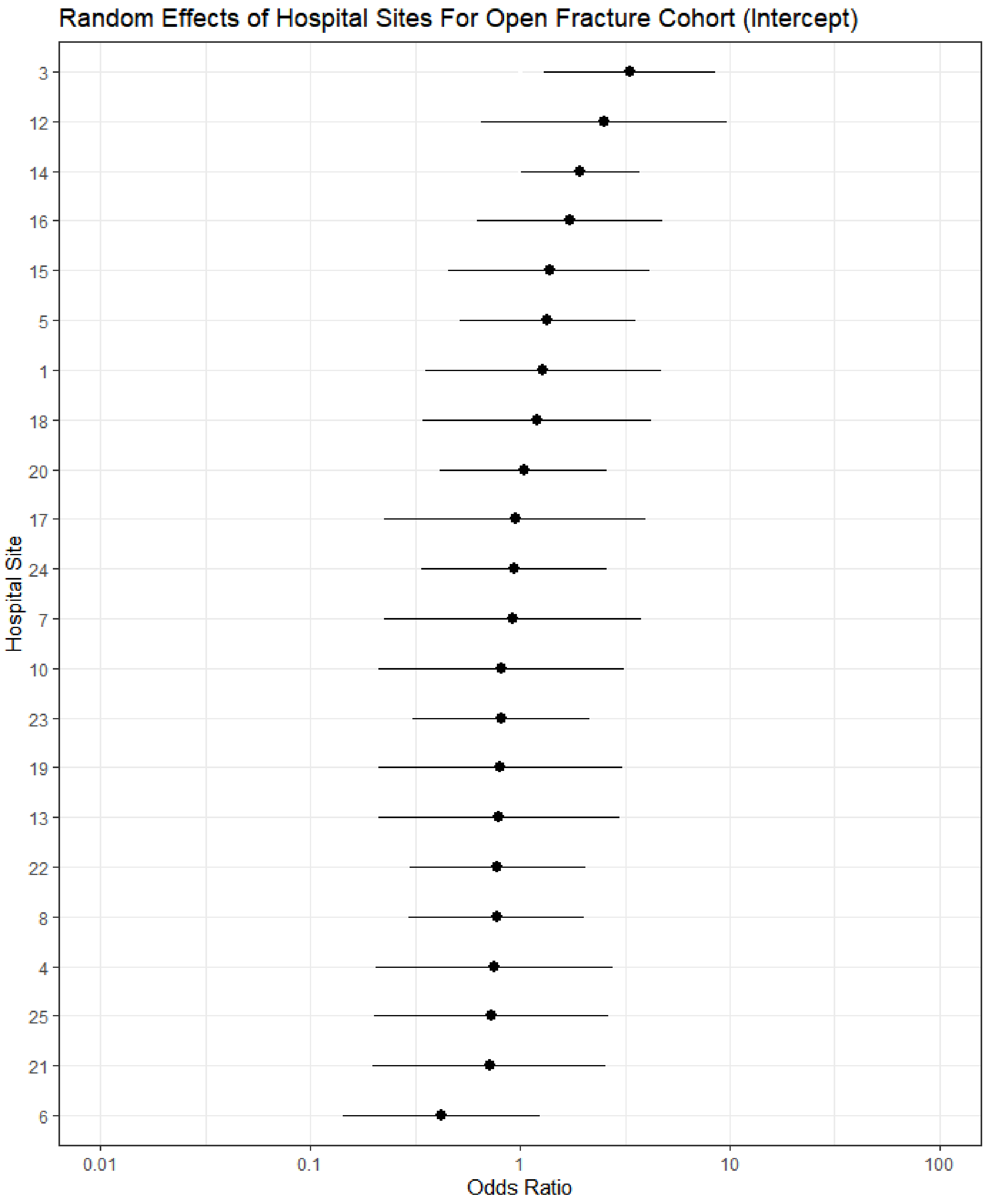

© 2021 Gitajn IL et al. *JAMA Health Forum.*

eFigure 2. Random Effects of Hospital Sites for Closed Fracture Cohort

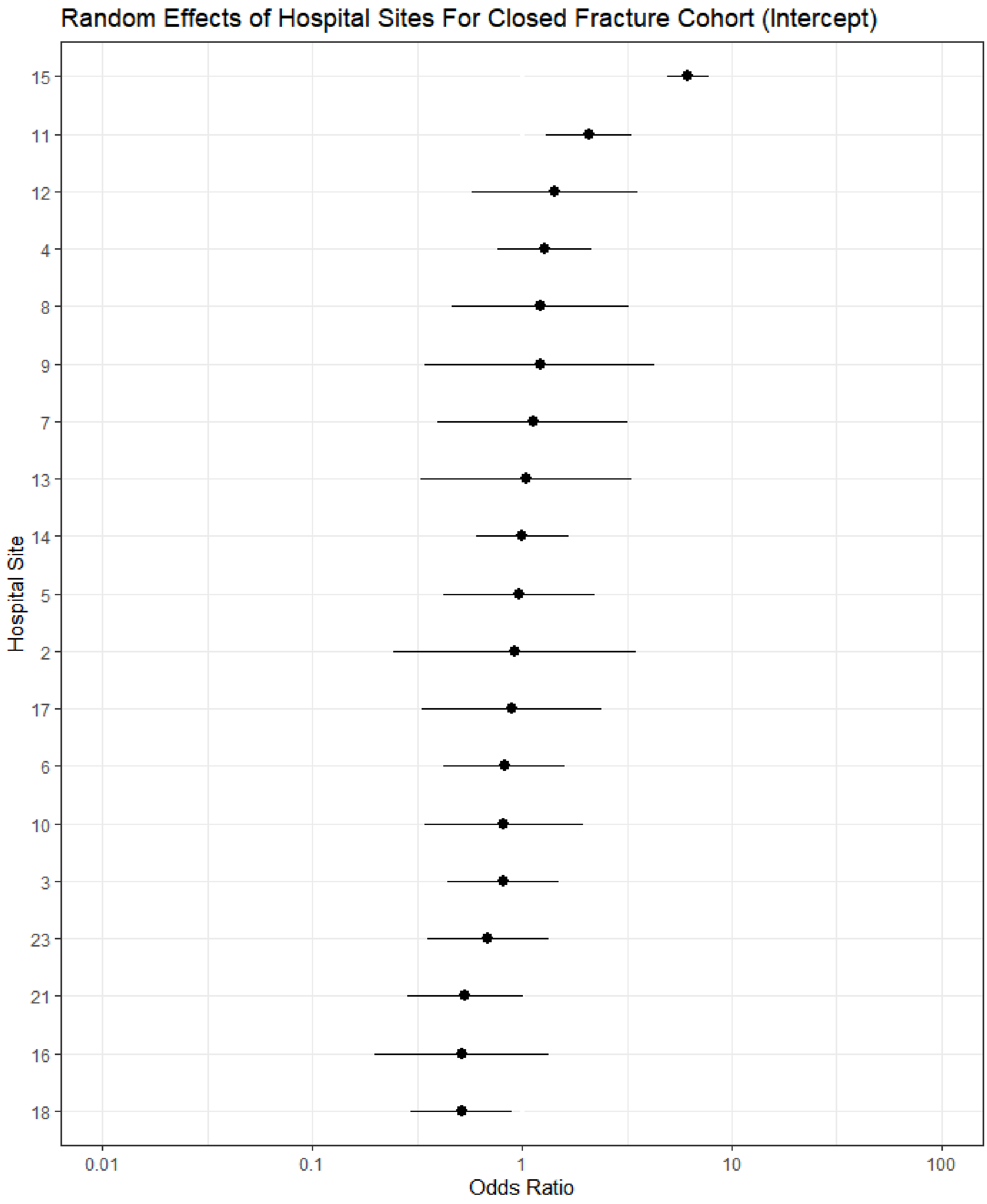

© 2021 Gitajn IL et al. *JAMA Health Forum.*

eFigure 3. Hip and Femur Fracture Interaction Plot

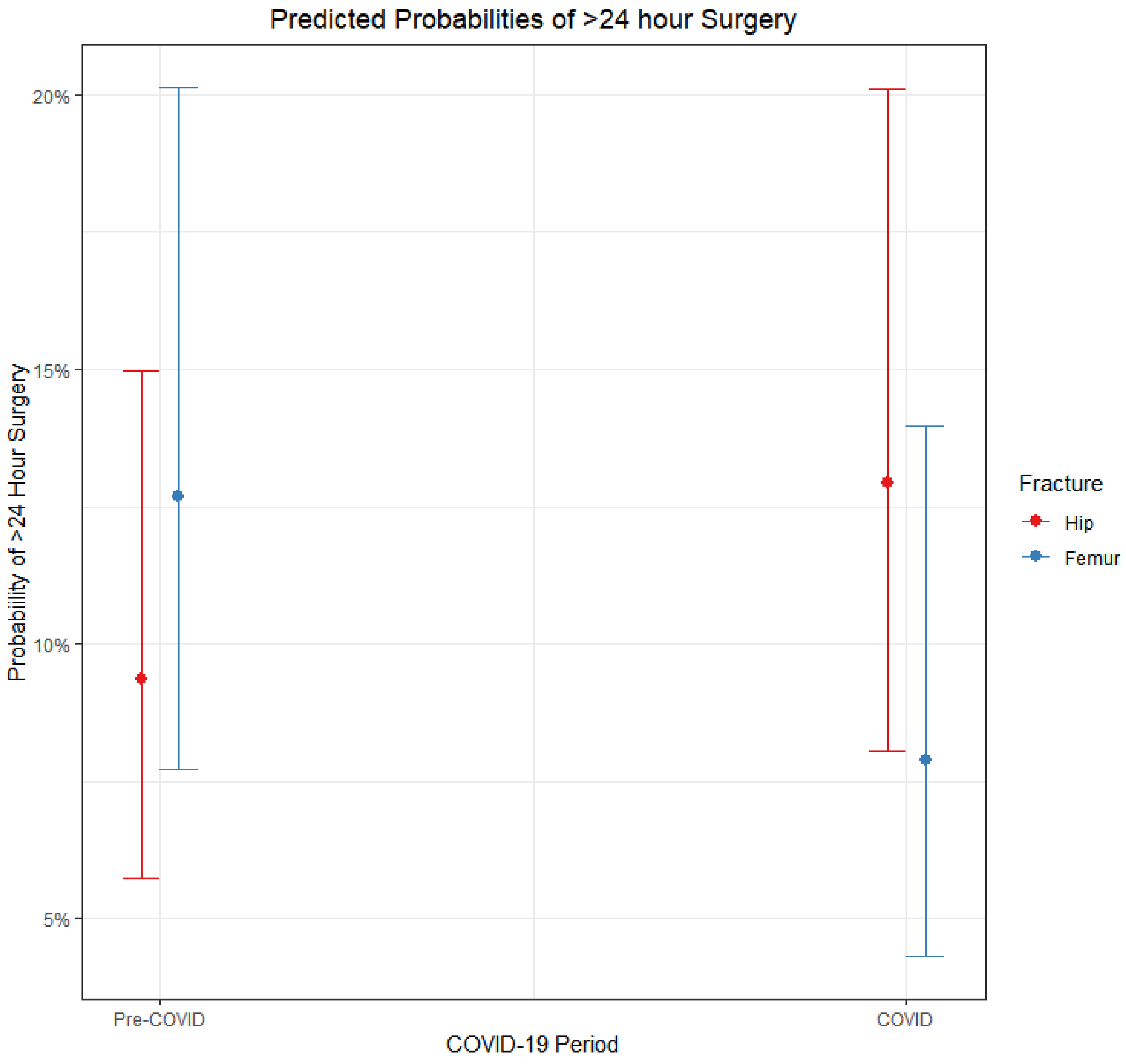

© 2021 Gitajn IL et al. *JAMA Health Forum.*

eFigure 4. Patient Enrollment Plot

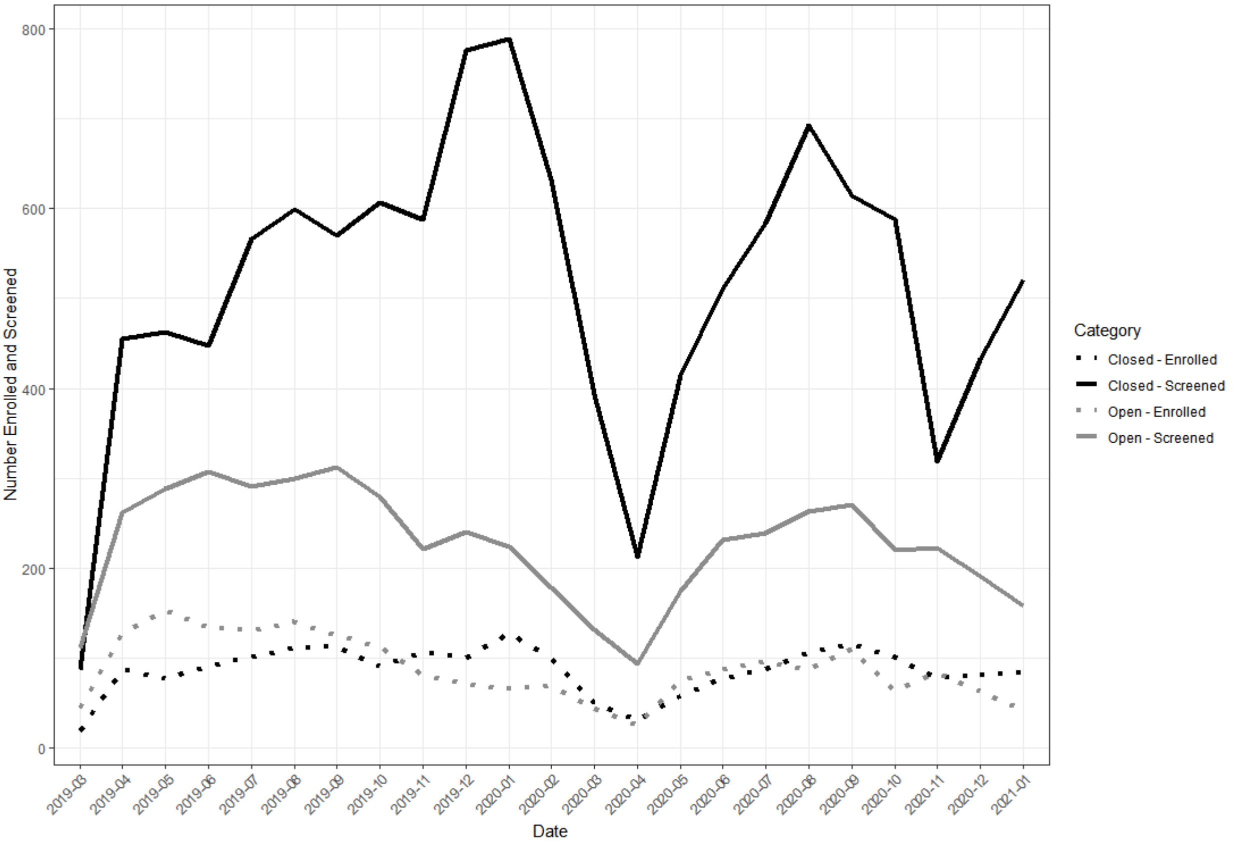

© 2021 Gitajn IL et al. *JAMA Health Forum.*
