# Supplementary material for: Association of COVID-19 With Achieving Time-to-Surgery Benchmarks in Patients With Musculoskeletal Trauma
Source: JAMA Health Forum. Author manuscript; Available in PMC 2022 Jan 18. (PMC8727030; doi:10.1001/jamahealthforum.2021.3460)
Supplement: Supplement 2 — PREP-IT Investigators Nonauthor Collaborators [file NIHMS1771809-supplement-Supplement_2.pdf]

Supplemental Online Content: Nonauthor Collaborators

*Indicates required information. Only first name, last name, and suffix will appear in PubMed.

*Group Name(s): PREP-IT Investigators							
***First Name and Middle Initial(s)**	***Last Name**	***Suffix (eg, Jr, III)**	Academic Degrees	Institution	Location (city, state/province, country)	Role or Contribution, eg, chair, principal investigator	Group (if more than 1 Group listed in the byline) and/or Subgroup (eg, Steering Committee)
Gerard P.	Slobogean		MD	University of Maryland School of Medicine	Baltimore, Maryland	Principal Investigator	Executive Committee
Sheila	Sprague		PhD	McMaster University	Hamilton, Ontario	Principal Investigator	Executive Committee
Jeffrey	Wells			Trauma Survivors Network	Falls Church, Virginia	Patient Representative	Executive Committee
Mohit	Bhandari		MD, PhD	McMaster University	Hamilton, Ontario	Principal Investigator	Executive Committee
Gerard P.	Slobogean		MD	University of Maryland School of Medicine	Baltimore, Maryland	Co-Chair	Steering Committee
Mohit	Bhandari		MD, PhD	McMaster University	Hamilton, Ontario	Co-Chair	Steering Committee
Sheila	Sprague		PhD	McMaster University	Hamilton, Ontario	Co-Chair	Steering Committee
Jean-Claude	D'Alleyrand			Walter Reed National Military Medical Center	Bethesda, MD	Committee Member	Steering Committee
Anthony D.	Harris			University of Maryland School of Medicine	Baltimore, MD	Committee Member	Steering Committee
Daniel C.	Mullins			University of Maryland	Baltimore, MD	Committee Member	Steering Committee
Lehana	Thabane			McMaster University	Hamilton, Ontario	Committee Member	Steering Committee
Jeffrey	Wells			Trauma Survivors Network	Falls Church, Virginia	Patient Partner	Steering Committee
Amber	Wood			Association of periOperative Registered Nurses	Denver, CO	Committee Member	Steering Committee
Gregory J.	Della Rocca			University of Missouri	Columbia, MO	Chair	Adjudication Committee
Anthony D.	Harris			University of Maryland School of Medicine	Baltimore, MD	Committee Member	Adjudication Committee
Joan	Hebden			University of Maryland	Baltimore, MD	Committee Member	Adjudication Committee
Kyle J.	Jeray			Greenville Health System	Greenville, SC	Committee Member	Adjudication Committee
Lucas	Marchand			University of Maryland	Baltimore, MD	Committee Member	Adjudication Committee

*Indicates required information. Only first name, last name, and suffix will appear in PubMed.

***First Name and Middle Initial(s)**	***Last Name**	***Suffix (eg, Jr, III)**	Academic Degrees	Institution	Location (city, state/province, country)	Role or Contribution, eg, chair, principal investigator	Group (if more than 1 Group listed in the byline) and/or Subgroup (eg, Steering Committee)
Lyndsay M.	O'Hara			University of Maryland School of Medicine	Baltimore, MD	Committee Member	Adjudication Committee
Robert	Zura			LSU Health	New Orleans, LA	Committee Member	Adjudication Committee
Michael J.	Gardner			Stanford University School of Medicine	Palo Alto, CA	Chair	Data and Safety Monitoring Committee
Jenna	Blasman				Kitchener, ON	Patient Representative	Data and Safety Monitoring Committee
Jonah	Davies			University of Washington	Seattle, WA	Committee Member	Data and Safety Monitoring Committee
Stephen	Liang			Washington University	St. Louis, MO	Committee Member	Data and Safety Monitoring Committee
Monica	Taljaard			Ottawa Hospital Research Institute	Ottawa, ON	Committee Member	Data and Safety Monitoring Committee
PJ	Devereaux			McMaster University	Hamilton, Ontario	Committee Member	Research Methodology Core
Gordon H.	Guyatt			McMaster University	Hamilton, Ontario	Committee Member	Research Methodology Core
Lehana	Thabane			McMaster University	Hamilton, Ontario	Committee Member	Research Methodology Core
Diane	Heels-Ansdell			McMaster University	Hamilton, Ontario	Committee Member	Research Methodology Core
Debra	Marvel				Baltimore, MD	Patient Representative	Patient Centred Outcomes Core
Jana	Palmer				Baltimore, MD	Patient Representative	Patient Centred Outcomes Core

Supplemental Online Content: Nonauthor Collaborators

*Indicates required information. Only first name, last name, and suffix will appear in PubMed.

*First Name and Middle Initial(s)	*Last Name	*Suffix (eg, Jr, III)	Academic Degrees	Institution	Location (city, state/province, country)	Role or Contribution, eg, chair, principal investigator	Group (if more than 1 Group listed in the byline) and/or Subgroup (eg, Steering Committee)
Jeffrey	Wells			Trauma Survivors Network	Baltimore, MD	Patient	Patient Centred Outcomes Core
Jeff	Friedrich			Slate Magazine	Washington, DC	Editor	Patient Centred Outcomes Core
Daniel C.	Mullins			University of Maryland	Baltimore, MD	Core Member	Patient Centred Outcomes Core
Nathan N.	O'Hara			University of Maryland School of Medicine	Baltimore, MD	Core Member	Patient Centred Outcomes Core
Frances	Grissom			Trauma Survivor Network	Baltimore, MD	Core Member	Patient Centred Outcomes Core
Gregory J.	Della Rocca			University of Missouri	Columbia, MO	Core Member	Orthopaedic Surgery Core
I. Leah	Gitajn			Dartmouth University	Hanover, NH	Core Member	Orthopaedic Surgery Core
Kyle J.	Jeray			Greenville Health System	Greenville, SC	Core Member	Orthopaedic Surgery Core
Saam	Morshed			San Francisco General Hospital	San Francisco, CA	Core Member	Orthopaedic Surgery Core
Robert V.	O'Toole			University of Maryland School of Medicine	Baltimore, MD	Core Member	Orthopaedic Surgery Core
Brad A.	Petrisor			Hamilton Health Sciences	Hamilton, Ontario	Core Member	Orthopaedic Surgery Core
Megan	Camara			R Adams Cowley Shock Trauma Center	Baltimore, MD	Core Member	Operating Room Core
Franca	Mossuto			Hamilton Health Sciences	Hamilton, Ontario	Core Member	Operating Room Core
Anthony D.	Harris			University of Maryland School of Medicine	Baltimore, MD	Core Member	Infectious Disease Core
Manjari G.	Joshi			University of Maryland School of Medicine	Baltimore, MD	Core Member	Infectious Disease Core
Jean-Claude	D'Alleyrand			Walter Reed National Military Medical Center	Bethesda, MD	Core Member	Military Core
Justin	Fowler			United States Army	USA	Core Member	Military Core

Supplemental Online Content: Nonauthor Collaborators

*Indicates required information. Only first name, last name, and suffix will appear in PubMed.

*First Name and Middle Initial(s)	*Last Name	*Suffix (eg, Jr, III)	Academic Degrees	Institution	Location (city, state/province, country)	Role or Contribution, eg, chair, principal investigator	Group (if more than 1 Group listed in the byline) and/or Subgroup (eg, Steering Committee)
Jessica	Rivera			San Antonio Military Medical Center	San Antonio, TX	Core Member	Military Core
Max	Talbot			Canadian Armed Forces	Montreal, QC	Core Member	Military Core
Sheila	Sprague		PhD	McMaster University	Hamilton, Ontario	Principal Investigator	McMaster University Methods Centre
Mohit	Bhandari		MD, PhD	McMaster University	Hamilton, Ontario	Principal Investigator	McMaster University Methods Centre
Shannon	Dodds			McMaster University	Hamilton, Ontario	Research Coordinator	McMaster University Methods Centre
Alisha	Garibaldi			McMaster University	Hamilton, Ontario	Research Coordinator	McMaster University Methods Centre
Silvia	Li		MSc	McMaster University	Hamilton, Ontario	Research Coordinator	McMaster University Methods Centre
Uyen	Nguyen		PhD	McMaster University	Hamilton, Ontario	Research Coordinator	McMaster University Methods Centre
David	Pogorzelski			McMaster University	Hamilton, Ontario	Research Coordinator	McMaster University Methods Centre
Alejandra	Rojas			McMaster University	Hamilton, Ontario	Research Coordinator	McMaster University Methods Centre
Taryn	Scott			McMaster University	Hamilton, Ontario	Research Coordinator	McMaster University Methods Centre
Gina	Del Fabbro		BPH	McMaster University	Hamilton, Ontario	Research Coordinator	McMaster University Methods Centre
Olivia P.	Szasz			McMaster University	Hamilton, Ontario	Research Assistant	McMaster University Methods Centre
Diane	Heels-Ansdell			McMaster University	Hamilton, Ontario	Statistician	McMaster University Methods Centre
Paula	McKay		BSc	McMaster University	Hamilton, Ontario	Manager	McMaster University Methods Centre
Gerard P.	Slobogean			University of Maryland School of Medicine	Baltimore, MD	Principal Investigator	University of Maryland School of Medicine Administrative Center

*Indicates required information. Only first name, last name, and suffix will appear in PubMed.

*First Name and Middle Initial(s)	*Last Name	*Suffix (eg, Jr, III)	Academic Degrees	Institution	Location (city, state/province, country)	Role or Contribution, eg, chair, principal investigator	Group (if more than 1 Group listed in the byline) and/or Subgroup (eg, Steering Committee)
Nathan N.	O'Hara			University of Maryland School of Medicine	Baltimore, MD	Manager	University of Maryland School of Medicine Administrative Center
Andrea	Howe			University of Maryland School of Medicine	Baltimore, MD	Project Manager	University of Maryland School of Medicine Administrative Center
Joshua	Rudnicki			University of Maryland School of Medicine	Baltimore, MD	Project Manager	University of Maryland School of Medicine Administrative Center
Haley	Demyanovich			University of Maryland School of Medicine	Baltimore, MD	Project Manager	University of Maryland School of Medicine Administrative Center
Kelly	Little			University of Maryland School of Medicine	Baltimore, MD	Financial Manager	University of Maryland School of Medicine Administrative Center
Adam	Boissonneault			University of Maryland School of Medicine	Baltimore, MD	Co-Investigator	University of Maryland School of Medicine Administrative Center
Daniel C.	Mullins			University of Maryland School of Pharmacy	Baltimore, MD	Executive Director	University of Maryland School of Pharmacy, The PATIENTS Program
Michelle	Medeiros			University of Maryland School of Pharmacy	Baltimore, MD	Director or Research	University of Maryland School of Pharmacy, The PATIENTS Program
Genevieve	Polk			University of Maryland School of Pharmacy	Baltimore, MD	Assistant Director, Dissemination and Research	University of Maryland School of Pharmacy, The PATIENTS Program
Eric	Kettering			University of Maryland School of Pharmacy	Baltimore, MD	Senior Instructional Technology and Dissemination Specialist	University of Maryland School of Pharmacy, The PATIENTS Program

*Indicates required information. Only first name, last name, and suffix will appear in PubMed.

***First Name and Middle Initial(s)**	***Last Name**	***Suffix (eg, Jr, III)**	Academic Degrees	Institution	Location (city, state/province, country)	Role or Contribution, eg, chair, principal investigator	Group (if more than 1 Group listed in the byline) and/or Subgroup (eg, Steering Committee)
Diamond	Hale			University of Maryland School of Pharmacy	Baltimore, MD	Project Manager	University of Maryland School of Pharmacy, The PATIENTS Program
Nirmen	Mahal			University of Maryland School of Pharmacy	Baltimore, MD	Program Specialist	University of Maryland School of Pharmacy, The PATIENTS Program
Robert V.	O'Toole			University of Maryland School of Medicine, R Adams Cowley Shock Trauma Center	Baltimore, MD	Co-Investigator	Lead Clinical Site (Aqueous-PREP and PREPARE)
Jean-Claude	D'Alleyrand			University of Maryland School of Medicine, R Adams Cowley Shock Trauma Center	Baltimore, MD	Co-Investigator	Lead Clinical Site (Aqueous-PREP and PREPARE)
Andrew	Eglseder			University of Maryland School of Medicine, R Adams Cowley Shock Trauma Center	Baltimore, MD	Co-Investigator	Lead Clinical Site (Aqueous-PREP and PREPARE)
Aaron	Johnson			University of Maryland School of Medicine, R Adams Cowley Shock Trauma Center	Baltimore, MD	Co-Investigator	Lead Clinical Site (Aqueous-PREP and PREPARE)
Christopher	Langhammer			University of Maryland School of Medicine, R Adams Cowley Shock Trauma Center	Baltimore, MD	Co-Investigator	Lead Clinical Site (Aqueous-PREP and PREPARE)
Christopher	Lebrun			University of Maryland School of Medicine, R Adams Cowley Shock Trauma Center	Baltimore, MD	Co-Investigator	Lead Clinical Site (Aqueous-PREP and PREPARE)
Theodore	Manson			University of Maryland School of Medicine, R Adams Cowley Shock Trauma Center	Baltimore, MD	Co-Investigator	Lead Clinical Site (Aqueous-PREP and PREPARE)
Jason	Nascone			University of Maryland School of Medicine, R Adams Cowley Shock Trauma Center	Baltimore, MD	Co-Investigator	Lead Clinical Site (Aqueous-PREP and PREPARE)

*Indicates required information. Only first name, last name, and suffix will appear in PubMed.

***First Name and Middle Initial(s)**	***Last Name**	***Suffix (eg, Jr, III)**	Academic Degrees	Institution	Location (city, state/province, country)	Role or Contribution, eg, chair, principal investigator	Group (if more than 1 Group listed in the byline) and/or Subgroup (eg, Steering Committee)
Ebrahim	Paryavi			University of Maryland School of Medicine, R Adams Cowley Shock Trauma Center	Baltimore, MD	Co-Investigator	Lead Clinical Site (Aqueous-PREP and PREPARE)
Raymond	Pensy			University of Maryland School of Medicine, R Adams Cowley Shock Trauma Center	Baltimore, MD	Co-Investigator	Lead Clinical Site (Aqueous-PREP and PREPARE)
Andrew	Pollak			University of Maryland School of Medicine, R Adams Cowley Shock Trauma Center	Baltimore, MD	Co-Investigator	Lead Clinical Site (Aqueous-PREP and PREPARE)
Marcus	Sciadini			University of Maryland School of Medicine, R Adams Cowley Shock Trauma Center	Baltimore, MD	Co-Investigator	Lead Clinical Site (Aqueous-PREP and PREPARE)
Gerard P.	Slobogean			University of Maryland School of Medicine, R Adams Cowley Shock Trauma Center	Baltimore, MD	Local Principal Investigator	Lead Clinical Site (Aqueous-PREP and PREPARE)
Yasmin	Degano			University of Maryland School of Medicine, R Adams Cowley Shock Trauma Center	Baltimore, MD	Research Assistant	Lead Clinical Site (Aqueous-PREP and PREPARE)
Haley K.	Demyanovich			University of Maryland School of Medicine, R Adams Cowley Shock Trauma Center	Baltimore, MD	Research Coordinator	Lead Clinical Site (Aqueous-PREP and PREPARE)
Andrea	Howe			University of Maryland School of Medicine, R Adams Cowley Shock Trauma Center	Baltimore, MD	Research Coordinator	Lead Clinical Site (Aqueous-PREP and PREPARE)
Nathan N.	O'Hara			University of Maryland School of Medicine, R Adams Cowley Shock Trauma Center	Baltimore, MD	Manager	Lead Clinical Site (Aqueous-PREP and PREPARE)
Katherine	Joseph			University of Maryland School of Medicine, R Adams Cowley Shock Trauma Center	Baltimore, MD	Research Coordinator	Lead Clinical Site (Aqueous-PREP and PREPARE)

*Indicates required information. Only first name, last name, and suffix will appear in PubMed.

***First Name and Middle Initial(s)**	***Last Name**	***Suffix (eg, Jr, III)**	Academic Degrees	Institution	Location (city, state/province, country)	Role or Contribution, eg, chair, principal investigator	Group (if more than 1 Group listed in the byline) and/or Subgroup (eg, Steering Committee)
Joshua	Rudnicki			University of Maryland School of Medicine, R Adams Cowley Shock Trauma Center	Baltimore, MD	Research Coordinator	Lead Clinical Site (Aqueous-PREP and PREPARE)
Megan	Camara			University of Maryland School of Medicine, R Adams Cowley Shock Trauma Center	Baltimore, MD	Research Coordinator	Lead Clinical Site (Aqueous-PREP and PREPARE)
Lucas	Marchand			University of Maryland School of Medicine, R Adams Cowley Shock Trauma Center	Baltimore, MD	Co-Investigator	Lead Clinical Site (Aqueous-PREP and PREPARE)
Heather	Phipps			University of Maryland School of Medicine, R Adams Cowley Shock Trauma Center	Baltimore, MD	Research Coordinator	Lead Clinical Site (Aqueous-PREP and PREPARE)
Eric	Hempen			University of Maryland School of Medicine, R Adams Cowley Shock Trauma Center	Baltimore, MD	Research Coordinator	Lead Clinical Site (Aqueous-PREP and PREPARE)
Brad A.	Petrisor			Hamilton Health Sciences – General Site	Hamilton, ON	Local Principal Investigator	Aqueous-PREP and PREPARE
Herman	Johal			Hamilton Health Sciences – General Site	Hamilton, ON	Local Principal Investigator	Aqueous-PREP and PREPARE
Bill	Ristevski			Hamilton Health Sciences – General Site	Hamilton, ON	Co-Investigator	Aqueous-PREP and PREPARE
Dale	Williams			Hamilton Health Sciences – General Site	Hamilton, ON	Co-Investigator	Aqueous-PREP and PREPARE
Matthew	Denkers			Hamilton Health Sciences – General Site	Hamilton, ON	Co-Investigator	Aqueous-PREP and PREPARE
Krishan	Rajaratnam			Hamilton Health Sciences – General Site	Hamilton, ON	Co-Investigator	Aqueous-PREP and PREPARE
Jamal	Al-Asiri			Hamilton Health Sciences – General Site	Hamilton, ON	Co-Investigator	Aqueous-PREP and PREPARE
Jordan	Leonard			Hamilton Health Sciences – General Site	Hamilton, ON	Research Coordinator	Aqueous-PREP and PREPARE

*Indicates required information. Only first name, last name, and suffix will appear in PubMed.

*First Name and Middle Initial(s)	*Last Name	*Suffix (eg, Jr, III)	Academic Degrees	Institution	Location (city, state/province, country)	Role or Contribution, eg, chair, principal investigator	Group (if more than 1 Group listed in the byline) and/or Subgroup (eg, Steering Committee)
Francesc A.	Marcano-Fernández			Hamilton Health Sciences – General Site	Hamilton, ON	Clinical Research Coordinator	Aqueous-PREP and PREPARE
Jodi	Gallant			Hamilton Health Sciences – General Site	Hamilton, ON	Clinical Research Coordinator	Aqueous-PREP and PREPARE
Federico	Persico			Hamilton Health Sciences – General Site	Hamilton, ON	Clinical Research Coordinator	Aqueous-PREP and PREPARE
Marko	Gjorgjievski			Hamilton Health Sciences – General Site	Hamilton, ON	Clinical Research Coordinator	Aqueous-PREP and PREPARE
Annie	George			Hamilton Health Sciences – General Site	Hamilton, ON	Clinical Research Coordinator	Aqueous-PREP and PREPARE
Sarah M.	McGaugh			Hamilton Health Sciences – General Site	Hamilton, ON	Clinical Research Coordinator	Aqueous-PREP and PREPARE
Kaitlyn	Pusztai			Hamilton Health Sciences – General Site	Hamilton, ON	Clinical Research Coordinator	Aqueous-PREP and PREPARE
Sarah	Piekarski			Hamilton Health Sciences – General Site	Hamilton, ON	Clinical Research Coordinator	Aqueous-PREP and PREPARE
Mavis	Lyons			Hamilton Health Sciences – General Site	Hamilton, ON	Clinical Research Coordinator	Aqueous-PREP and PREPARE
Julia	Gennaccaro			Hamilton Health Sciences – General Site	Hamilton, ON	Clinical Research Coordinator	Aqueous-PREP and PREPARE
Roman N.	Natoli			IU Health Methodist Hospital	Indianapolis, IN	Local Principal Investigator	Aqueous-PREP and PREPARE
Greg E.	Gaski			IU Health Methodist Hospital	Indianapolis, IN	Co-Investigator	Aqueous-PREP and PREPARE
Todd O.	McKinley			IU Health Methodist Hospital	Indianapolis, IN	Co-Investigator	Aqueous-PREP and PREPARE
Walter W.	Virkus			IU Health Methodist Hospital	Indianapolis, IN	Co-Investigator	Aqueous-PREP and PREPARE
Anthony T.	Sorkin			IU Health Methodist Hospital	Indianapolis, IN	Co-Investigator	Aqueous-PREP and PREPARE
Jan P.	Szatkowski			IU Health Methodist Hospital	Indianapolis, IN	Co-Investigator	Aqueous-PREP and PREPARE

Supplemental Online Content: Nonauthor Collaborators

*Indicates required information. Only first name, last name, and suffix will appear in PubMed.

*First Name and Middle Initial(s)	*Last Name	*Suffix (eg, Jr, III)	Academic Degrees	Institution	Location (city, state/province, country)	Role or Contribution, eg, chair, principal investigator	Group (if more than 1 Group listed in the byline) and/or Subgroup (eg, Steering Committee)
Joseph R.	Baele			IU Health Methodist Hospital	Indianapolis, IN	Co-Investigator	Aqueous-PREP and PREPARE
Brian H.	Mullis			IU Health Methodist Hospital	Indianapolis, IN	Co-Investigator	Aqueous-PREP and PREPARE
Yohan	Jang						
Lauren C.	Hill			IU Health Methodist Hospital	Indianapolis, IN	Research Coordinator	Aqueous-PREP and PREPARE
Andrea	Hudgins			IU Health Methodist Hospital	Indianapolis, IN	Research Coordinator	Aqueous-PREP and PREPARE
Courteney L.	Fentz			IU Health Methodist Hospital	Indianapolis, IN	Research Coordinator	Aqueous-PREP and PREPARE
Maricela M.	Diaz			IU Health Methodist Hospital	Indianapolis, IN	Research Coordinator	Aqueous-PREP and PREPARE
Katelyn M.	Garst			IU Health Methodist Hospital	Indianapolis, IN	Research Coordinator	Aqueous-PREP and PREPARE
Emma W.	Denari			IU Health Methodist Hospital	Indianapolis, IN	Research Coordinator	Aqueous-PREP and PREPARE
Patrick	Osborn			San Antonio Military Medical Center	San Antonio, TX	Local Principal Investigator	Aqueous-PREP and PREPARE
Justin	Fowler			San Antonio Military Medical Center	San Antonio, TX	Co-Investigator	Aqueous-PREP and PREPARE
Sarah	Pierrie			San Antonio Military Medical Center	San Antonio, TX	Local Principal Investigator/Co-Investigator	Aqueous-PREP and PREPARE
Eric	Martinez			San Antonio Military Medical Center	San Antonio, TX	Research Coordinator	Aqueous-PREP and PREPARE
Joseph	Kimmel			San Antonio Military Medical Center	San Antonio, TX	Research Coordinator	Aqueous-PREP and PREPARE
Kyle J.	Jeray			Prisma Health - Upstate	Greenville, SC	Local Principal Investigator	Aqueous-PREP and PREPARE
John D.	Adams			Prisma Health - Upstate	Greenville, SC	Co-Investigator	Aqueous-PREP and PREPARE

*Indicates required information. Only first name, last name, and suffix will appear in PubMed.

*First Name and Middle Initial(s)	*Last Name	*Suffix (eg, Jr, III)	Academic Degrees	Institution	Location (city, state/province, country)	Role or Contribution, eg, chair, principal investigator	Group (if more than 1 Group listed in the byline) and/or Subgroup (eg, Steering Committee)
Michael L.	Beckish			Prisma Health - Upstate	Greenville, SC	Co-Investigator	Aqueous-PREP and PREPARE
Christopher C.	Bray			Prisma Health - Upstate	Greenville, SC	Co-Investigator	Aqueous-PREP and PREPARE
Timothy R.	Brown			Prisma Health - Upstate	Greenville, SC	Co-Investigator	Aqueous-PREP and PREPARE
Andrew W.	Cross			Prisma Health - Upstate	Greenville, SC	Co-Investigator	Aqueous-PREP and PREPARE
Timothy	Dew			Prisma Health - Upstate	Greenville, SC	Co-Investigator	Aqueous-PREP and PREPARE
Gregory K.	Faucher			Prisma Health - Upstate	Greenville, SC	Co-Investigator	Aqueous-PREP and PREPARE
Richard W.	Gurich	Jr.		Prisma Health - Upstate	Greenville, SC	Co-Investigator	Aqueous-PREP and PREPARE
David E.	Lazarus			Prisma Health - Upstate	Greenville, SC	Co-Investigator	Aqueous-PREP and PREPARE
S. John	Millon			Prisma Health - Upstate	Greenville, SC	Co-Investigator	Aqueous-PREP and PREPARE
M. Jason	Palmer			Prisma Health - Upstate	Greenville, SC	Co-Investigator	Aqueous-PREP and PREPARE
Scott E.	Porter			Prisma Health - Upstate	Greenville, SC	Co-Investigator	Aqueous-PREP and PREPARE
Thomas M.	Schaller			Prisma Health - Upstate	Greenville, SC	Co-Investigator	Aqueous-PREP and PREPARE
Michael S.	Sridhar			Prisma Health - Upstate	Greenville, SC	Co-Investigator	Aqueous-PREP and PREPARE
John L.	Sanders			Prisma Health - Upstate	Greenville, SC	Co-Investigator	Aqueous-PREP and PREPARE
L. Edwin	Rudisill	Jr.		Prisma Health - Upstate	Greenville, SC	Co-Investigator	Aqueous-PREP and PREPARE
Michael J.	Garitty			Prisma Health - Upstate	Greenville, SC	Co-Investigator	Aqueous-PREP and PREPARE

*Indicates required information. Only first name, last name, and suffix will appear in PubMed.

*First Name and Middle Initial(s)	*Last Name	*Suffix (eg, Jr, III)	Academic Degrees	Institution	Location (city, state/province, country)	Role or Contribution, eg, chair, principal investigator	Group (if more than 1 Group listed in the byline) and/or Subgroup (eg, Steering Committee)
Andrew S.	Poole			Prisma Health - Upstate	Greenville, SC	Co-Investigator	Aqueous-PREP and PREPARE
Michael L.	Sims			Prisma Health - Upstate	Greenville, SC	Co-Investigator	Aqueous-PREP and PREPARE
Robert M.	Carlisle	II		Prisma Health - Upstate	Greenville, SC	Co-Investigator	Aqueous-PREP and PREPARE
Erin	Adams-Hofer			Prisma Health - Upstate	Greenville, SC	Co-Investigator	Aqueous-PREP and PREPARE
Brandon S.	Huggins			Prisma Health - Upstate	Greenville, SC	Co-Investigator	Aqueous-PREP and PREPARE
Michael D.	Hunter			Prisma Health - Upstate	Greenville, SC	Co-Investigator	Aqueous-PREP and PREPARE
William A.	Marshall			Prisma Health - Upstate	Greenville, SC	Co-Investigator	Aqueous-PREP and PREPARE
Shea	Bielby Ray			Prisma Health - Upstate	Greenville, SC	Co-Investigator	Aqueous-PREP and PREPARE
Cory D.	Smith			Prisma Health - Upstate	Greenville, SC	Co-Investigator	Aqueous-PREP and PREPARE
Kyle M.	Altman			Prisma Health - Upstate	Greenville, SC	Co-Investigator	Aqueous-PREP and PREPARE
Julia C.	Bedard			Prisma Health - Upstate	Greenville, SC	Co-Investigator	Aqueous-PREP and PREPARE
Markus F.	Loeffler			Prisma Health - Upstate	Greenville, SC	Co-Investigator	Aqueous-PREP and PREPARE
Erin R.	Pichiotino			Prisma Health - Upstate	Greenville, SC	Co-Investigator	Aqueous-PREP and PREPARE
Austin A.	Cole			Prisma Health - Upstate	Greenville, SC	Co-Investigator	Aqueous-PREP and PREPARE
Ethan J.	Maltz			Prisma Health - Upstate	Greenville, SC	Co-Investigator	Aqueous-PREP and PREPARE
Wesley	Parker			Prisma Health - Upstate	Greenville, SC	Co-Investigator	Aqueous-PREP and PREPARE

*Indicates required information. Only first name, last name, and suffix will appear in PubMed.

*First Name and Middle Initial(s)	*Last Name	*Suffix (eg, Jr, III)	Academic Degrees	Institution	Location (city, state/province, country)	Role or Contribution, eg, chair, principal investigator	Group (if more than 1 Group listed in the byline) and/or Subgroup (eg, Steering Committee)
T. Bennett	Ramsey			Prisma Health - Upstate	Greenville, SC	Co-Investigator	Aqueous-PREP and PREPARE
Alex	Burnikel			Prisma Health - Upstate	Greenville, SC	Co-Investigator	Aqueous-PREP and PREPARE
Michael	Colello			Prisma Health - Upstate	Greenville, SC	Co-Investigator	Aqueous-PREP and PREPARE
Russel	Stewart			Prisma Health - Upstate	Greenville, SC	Co-Investigator	Aqueous-PREP and PREPARE
Jeremy	Wise			Prisma Health - Upstate	Greenville, SC	Co-Investigator	Aqueous-PREP and PREPARE
M. Christian	Moody			Prisma Health - Upstate	Greenville, SC	Co-Investigator	Aqueous-PREP and PREPARE
Matthew	Anderson			Prisma Health - Upstate	Greenville, SC	Co-Investigator	Aqueous-PREP and PREPARE
Joshua	Eskew			Prisma Health - Upstate	Greenville, SC	Co-Investigator	Aqueous-PREP and PREPARE
Benjamin	Judkins			Prisma Health - Upstate	Greenville, SC	Co-Investigator	Aqueous-PREP and PREPARE
James M.	Miller			Prisma Health - Upstate	Greenville, SC	Co-Investigator	Aqueous-PREP and PREPARE
Stephanie L.	Tanner			Prisma Health - Upstate	Greenville, SC	Research Coordinator	Aqueous-PREP and PREPARE
Rebecca G.	Snider			Prisma Health - Upstate	Greenville, SC	Research Coordinator	Aqueous-PREP and PREPARE
Christine E.	Townsend			Prisma Health - Upstate	Greenville, SC	Research Coordinator	Aqueous-PREP and PREPARE
Kayla H.	Pham			Prisma Health - Upstate	Greenville, SC	Research Coordinator	Aqueous-PREP and PREPARE
Abigail	Martin			Prisma Health - Upstate	Greenville, SC	Research Coordinator	Aqueous-PREP and PREPARE
Emily	Robertson			Prisma Health - Upstate	Greenville, SC	Research Coordinator	Aqueous-PREP and PREPARE

Supplemental Online Content: Nonauthor Collaborators

*Indicates required information. Only first name, last name, and suffix will appear in PubMed.

***First Name and Middle Initial(s)**	***Last Name**	***Suffix (eg, Jr, III)**	Academic Degrees	Institution	Location (city, state/province, country)	Role or Contribution, eg, chair, principal investigator	Group (if more than 1 Group listed in the byline) and/or Subgroup (eg, Steering Committee)
John Wilson	Skyes			Prisma Health - Upstate	Greenville, SC	Research Coordinator	Aqueous-PREP and PREPARE
Saam	Morshed			University of California, San Francisco	San Francisco, CA	Local Principal Investigator	Aqueous-PREP and PREPARE
Utku	Kandemir			University of California, San Francisco	San Francisco, CA	Co-Investigator	Aqueous-PREP and PREPARE
Meir	Marmor			University of California, San Francisco	San Francisco, CA	Local Principal Investigator	Aqueous-PREP and PREPARE
Amir	Matityahu			University of California, San Francisco	San Francisco, CA	Co-Investigator	Aqueous-PREP and PREPARE
R. Trigg	McClellan			University of California, San Francisco	San Francisco, CA	Co-Investigator	Aqueous-PREP and PREPARE
Eric	Meinberg			University of California, San Francisco	San Francisco, CA	Co-Investigator	Aqueous-PREP and PREPARE
Theodore	Miclau			University of California, San Francisco	San Francisco, CA	Co-Investigator	Aqueous-PREP and PREPARE
David	Shearer			University of California, San Francisco	San Francisco, CA	Co-Investigator	Aqueous-PREP and PREPARE
Paul	Toogood			University of California, San Francisco	San Francisco, CA	Co-Investigator	Aqueous-PREP and PREPARE
Anthony	Ding			University of California, San Francisco	San Francisco, CA	Co-Investigator	Aqueous-PREP and PREPARE
Erin	Donohue			University of California, San Francisco	San Francisco, CA	Co-Investigator	Aqueous-PREP and PREPARE
Jothi	Murali			University of California, San Francisco	San Francisco, CA	Co-Investigator	Aqueous-PREP and PREPARE
Ashraf	El Naga			University of California, San Francisco	San Francisco, CA	Co-Investigator	Aqueous-PREP and PREPARE
Jennifer	Tangtiphaiboontana			University of California, San Francisco	San Francisco, CA	Co-Investigator	Aqueous-PREP and PREPARE
Tigist	Belaye			University of California, San Francisco	San Francisco, CA	Research Coordinator	Aqueous-PREP and PREPARE

*Indicates required information. Only first name, last name, and suffix will appear in PubMed.

*First Name and Middle Initial(s)	*Last Name	*Suffix (eg, Jr, III)	Academic Degrees	Institution	Location (city, state/province, country)	Role or Contribution, eg, chair, principal investigator	Group (if more than 1 Group listed in the byline) and/or Subgroup (eg, Steering Committee)
Eleni	Berhaneselase			University of California, San Francisco	San Francisco, CA	Research Coordinator	Aqueous-PREP and PREPARE
Alexandra	Paul			University of California, San Francisco	San Francisco, CA	Research Coordinator	Aqueous-PREP and PREPARE
Kartik	Garg			University of California, San Francisco	San Francisco, CA	Research Coordinator	Aqueous-PREP and PREPARE
Dimitry	Pokhvashchev			University of California, San Francisco	San Francisco, CA	Research Coordinator	Aqueous-PREP and PREPARE
Joshua L.	Gary			McGovern Medical School at UTHealth Houston	Houston, TX	Local Principal Investigator	Aqueous-PREP
Stephanie J.	Warner			McGovern Medical School at UTHealth Houston	Houston, TX	Co-Investigator	Aqueous-PREP
John W.	Munz			McGovern Medical School at UTHealth Houston	Houston, TX	Co-Investigator	Aqueous-PREP
Andrew M.	Choo			McGovern Medical School at UTHealth Houston	Houston, TX	Co-Investigator	Aqueous-PREP
Timothy S.	Schor			McGovern Medical School at UTHealth Houston	Houston, TX	Co-Investigator	Aqueous-PREP
Milton L. "Chip"	Routt			McGovern Medical School at UTHealth Houston	Houston, TX	Co-Investigator	Aqueous-PREP
Mayank	Rao			McGovern Medical School at UTHealth Houston	Houston, TX	Research Coordinator	Aqueous-PREP
Guillermo	Pechero			McGovern Medical School at UTHealth Houston	Houston, TX	Research Coordinator	Aqueous-PREP
Adam	Miller			McGovern Medical School at UTHealth Houston	Houston, TX	Research Coordinator	Aqueous-PREP
Michael	Kutzler			McGovern Medical School at UTHealth Houston	Houston, TX	Research Coordinator	Aqueous-PREP
Jennifer E.	Hagen			University of Florida	Gainesville, FL	Local Principal Investigator	Aqueous-PREP
Matthew	Patrick			University of Florida	Gainesville, FL	Co-Investigator	Aqueous-PREP
Richard	Vlasak			University of Florida	Gainesville, FL	Co-Investigator	Aqueous-PREP

*Indicates required information. Only first name, last name, and suffix will appear in PubMed.

*First Name and Middle Initial(s)	*Last Name	*Suffix (eg, Jr, III)	Academic Degrees	Institution	Location (city, state/province, country)	Role or Contribution, eg, chair, principal investigator	Group (if more than 1 Group listed in the byline) and/or Subgroup (eg, Steering Committee)
Thomas	Krupko			University of Florida	Gainesville, FL	Co-Investigator	Aqueous-PREP
Kalia	Sadasivan			University of Florida	Gainesville, FL	Co-Investigator	Aqueous-PREP
Michael	Talerico			University of Florida	Gainesville, FL	Co-Investigator	Aqueous-PREP
Marybeth	Horodyski			University of Florida	Gainesville, FL	Co-Investigator	Aqueous-PREP
Chris	Koenig			University of Florida	Gainesville, FL	Research Coordinator	Aqueous-PREP
Daniel	Bailey			University of Florida	Gainesville, FL	Research Coordinator	Aqueous-PREP
Daniel	Wentworth			University of Florida	Gainesville, FL	Research Coordinator	Aqueous-PREP
Chi	Van			University of Florida	Gainesville, FL	Research Coordinator	Aqueous-PREP
Justin	Schwartz			University of Florida	Gainesville, FL	Research Coordinator	Aqueous-PREP
Marissa	Pazik			University of Florida	Gainesville, FL	Research Coordinator	Aqueous-PREP
Niloofar	Dehghan			University of Florida	Gainesville, FL	Local Principal Investigator	Aqueous-PREP
Clifford B.	Jones			The CORE Institute	Phoenix, AZ	Local Principal Investigator	Aqueous-PREP
J. Tracy	Watson			The CORE Institute	Phoenix, AZ	Co-Investigator	Aqueous-PREP
Michael	McKee			The CORE Institute	Phoenix, AZ	Co-Investigator	Aqueous-PREP
Ammar	Karim			The CORE Institute	Phoenix, AZ	Co-Investigator	Aqueous-PREP
Michael	Talerico			The CORE Institute	Phoenix, AZ	Co-Investigator	Aqueous-PREP
Debra L.	Sietsema			The CORE Institute	Phoenix, AZ	Research Coordinator	Aqueous-PREP
Alyse	Williams			The CORE Institute	Phoenix, AZ	Research Coordinator	Aqueous-PREP
Tayler	Dykes			The CORE Institute	Phoenix, AZ	Research Coordinator	Aqueous-PREP
William T.	Obremsky			Vanderbilt Medical Center	Nashville, TN	Local Principal Investigator	Aqueous-PREP
Amir A.	Jahangir			Vanderbilt Medical Center	Nashville, TN	Co-Investigator	Aqueous-PREP
Manish	Sethi			Vanderbilt Medical Center	Nashville, TN	Co-Investigator	Aqueous-PREP
Robert	Boyce			Vanderbilt Medical Center	Nashville, TN	Co-Investigator	Aqueous-PREP
Philip	Mitchell			Vanderbilt Medical Center	Nashville, TN	Co-Investigator	Aqueous-PREP
Daniel J.	Stinner			Vanderbilt Medical Center	Nashville, TN	Co-Investigator	Aqueous-PREP
Karen	Trochez			Vanderbilt Medical Center	Nashville, TN	Research Coordinator	Aqueous-PREP
Andres	Rodriguez			Vanderbilt Medical Center	Nashville, TN	Research Coordinator	Aqueous-PREP
Vamshi	Gajari			Vanderbilt Medical Center	Nashville, TN	Research Coordinator	Aqueous-PREP
Elsa	Rodriguez			Vanderbilt Medical Center	Nashville, TN	Research Coordinator	Aqueous-PREP

Supplemental Online Content: Nonauthor Collaborators

*Indicates required information. Only first name, last name, and suffix will appear in PubMed.

*First Name and Middle Initial(s)	*Last Name	*Suffix (eg, Jr, III)	Academic Degrees	Institution	Location (city, state/province, country)	Role or Contribution, eg, chair, principal investigator	Group (if more than 1 Group listed in the byline) and/or Subgroup (eg, Steering Committee)
Charles	Pritchett			Vanderbilt Medical Center	Nashville, TN	Research Coordinator	Aqueous-PREP
Natalie	Hogan			Vanderbilt Medical Center	Nashville, TN	Research Coordinator	Aqueous-PREP
A. Fidel	Moreno			Vanderbilt Medical Center	Nashville, TN	Research Coordinator	Aqueous-PREP
Christina	Boulton			Banner University Medical Center – Tucson	Tucson, AZ	Local Principal Investigator	Aqueous-PREP
Jason	Lowe			Banner University Medical Center – Tucson	Tucson, AZ	Co-Investigator	Aqueous-PREP
Jason	Wild			Banner University Medical Center – Tucson	Tucson, AZ	Co-Investigator	Aqueous-PREP
John T.	Ruth			Banner University Medical Center – Tucson	Tucson, AZ	Co-Investigator	Aqueous-PREP
Michel	Taylor			Banner University Medical Center – Tucson	Tucson, AZ	Co-Investigator	Aqueous-PREP
Brad	Askam			Banner University Medical Center – Tucson	Tucson, AZ	Co-Investigator	Aqueous-PREP
Andrea	Seach			Banner University Medical Center – Tucson	Tucson, AZ	Manager	Aqueous-PREP
Sabina	Saeed			Banner University Medical Center – Tucson	Tucson, AZ	Research Coordinator	Aqueous-PREP
Hunter	Culbert			Banner University Medical Center – Tucson	Tucson, AZ	Research Coordinator	Aqueous-PREP
Alejandro	Cruz			Banner University Medical Center – Tucson	Tucson, AZ	Research Coordinator	Aqueous-PREP
Thomas	Knapp			Banner University Medical Center – Tucson	Tucson, AZ	Research Coordinator	Aqueous-PREP
Colin	Hurkett			Banner University Medical Center – Tucson	Tucson, AZ	Research Coordinator	Aqueous-PREP
Maya	Lowney			Banner University Medical Center – Tucson	Tucson, AZ	Research Coordinator	Aqueous-PREP
Breanna	Featherston			Banner University Medical Center – Tucson	Tucson, AZ	Research Coordinator	Aqueous-PREP

*Indicates required information. Only first name, last name, and suffix will appear in PubMed.

*First Name and Middle Initial(s)	*Last Name	*Suffix (eg, Jr, III)	Academic Degrees	Institution	Location (city, state/province, country)	Role or Contribution, eg, chair, principal investigator	Group (if more than 1 Group listed in the byline) and/or Subgroup (eg, Steering Committee)
Michael	Prayson			Wright State University	Dayton, OH	Local Principal Investigator	Aqueous-PREP
Indresh	Venkatarayappa			Wright State University	Dayton, OH	Co-Investigator	Aqueous-PREP
Brandon	Horne			Wright State University	Dayton, OH	Co-Investigator	Aqueous-PREP
Jennifer	Jerele			Wright State University	Dayton, OH	Co-Investigator	Aqueous-PREP
Linda	Clark			Wright State University	Dayton, OH	Research Coordinator	Aqueous-PREP
Francesc	Marcano-Fernández			Hospital Universitari Parc Tauli	Barcelona, Spain	Local Principal Investigator	Aqueous-PREP
Montsant	Jornet-Gibert			Hospital Universitari Parc Tauli	Barcelona, Spain	Co-Investigator	Aqueous-PREP
Laia	Martinez-Carreres			Hospital Universitari Parc Tauli	Barcelona, Spain	Research Coordinator	Aqueous-PREP
David	Marti-Garin			Hospital Universitari Parc Tauli	Barcelona, Spain	Research Coordinator	Aqueous-PREP
Jorge	Serrano-Sanz			Hospital Universitari Parc Tauli	Barcelona, Spain	Research Coordinator	Aqueous-PREP
Joel	Sanchez-Fernandez			Hospital Universitari Parc Tauli	Barcelona, Spain	Research Coordinator	Aqueous-PREP
Matsuyama	Sanz-Molero			Hospital Universitari Parc Tauli	Barcelona, Spain	Research Coordinator	Aqueous-PREP
Alejandro	Carballo			Hospital Universitari Parc Tauli	Barcelona, Spain	Research Coordinator	Aqueous-PREP
Xavier	Pelfort			Hospital Universitari Parc Tauli	Barcelona, Spain	Research Coordinator	Aqueous-PREP
Francesc	Acerboni-Flores			Hospital Universitari Parc Tauli	Barcelona, Spain	Research Coordinator	Aqueous-PREP
Anna	Alavedra-Massana			Hospital Universitari Parc Tauli	Barcelona, Spain	Research Coordinator	Aqueous-PREP
Neus	Anglada-Torres			Hospital Universitari Parc Tauli	Barcelona, Spain	Research Coordinator	Aqueous-PREP
Alexandre	Berenguer			Hospital Universitari Parc Tauli	Barcelona, Spain	Research Coordinator	Aqueous-PREP
Jaume	Camara-Cabrera			Hospital Universitari Parc Tauli	Barcelona, Spain	Research Coordinator	Aqueous-PREP
Ariadna	Caparros-Garcia			Hospital Universitari Parc Tauli	Barcelona, Spain	Research Coordinator	Aqueous-PREP
Ferran	Fillat-Goma			Hospital Universitari Parc Tauli	Barcelona, Spain	Research Coordinator	Aqueous-PREP
Ruben	Fuentes-Lopez			Hospital Universitari Parc Tauli	Barcelona, Spain	Research Coordinator	Aqueous-PREP
Ramona	Garcia-Rodriguez			Hospital Universitari Parc Tauli	Barcelona, Spain	Research Coordinator	Aqueous-PREP
Nuria	Gimeno-Calavia			Hospital Universitari Parc Tauli	Barcelona, Spain	Research Coordinator	Aqueous-PREP
Guillem	Graells-Alonso			Hospital Universitari Parc Tauli	Barcelona, Spain	Research Coordinator	Aqueous-PREP
Marta	Martinez-Alvarez			Hospital Universitari Parc Tauli	Barcelona, Spain	Research Coordinator	Aqueous-PREP

*Indicates required information. Only first name, last name, and suffix will appear in PubMed.

*First Name and Middle Initial(s)	*Last Name	*Suffix (eg, Jr, III)	Academic Degrees	Institution	Location (city, state/province, country)	Role or Contribution, eg, chair, principal investigator	Group (if more than 1 Group listed in the byline) and/or Subgroup (eg, Steering Committee)
Patricia	Martinez-Grau			Hospital Universitari Parc Tauli	Barcelona, Spain	Research Coordinator	Aqueous-PREP
Raul	Pellejero-Garcia			Hospital Universitari Parc Tauli	Barcelona, Spain	Research Coordinator	Aqueous-PREP
Ona	Rafols-Perramon			Hospital Universitari Parc Tauli	Barcelona, Spain	Research Coordinator	Aqueous-PREP
Juan Manuel	Penalver			Hospital Universitari Parc Tauli	Barcelona, Spain	Research Coordinator	Aqueous-PREP
Monica Salomo	Domenech			Hospital Universitari Parc Tauli	Barcelona, Spain	Research Coordinator	Aqueous-PREP
Albert	Soler-Cano			Hospital Universitari Parc Tauli	Barcelona, Spain	Research Coordinator	Aqueous-PREP
Aldo	Velasco-Barrera			Hospital Universitari Parc Tauli	Barcelona, Spain	Research Coordinator	Aqueous-PREP
Christian	Yela-Verdú			Hospital Universitari Parc Tauli	Barcelona, Spain	Research Coordinator	Aqueous-PREP
Mercedes	Bueno-Ruiz			Hospital Universitari Parc Tauli	Barcelona, Spain	Research Coordinator	Aqueous-PREP
Estrella	Sánchez-Palomino			Hospital Universitari Parc Tauli	Barcelona, Spain	Research Coordinator	Aqueous-PREP
Vito	Andriola			Hospital Universitari Parc Tauli	Barcelona, Spain	Research Coordinator	Aqueous-PREP
Matilde	Molina-Corbacho			Hospital Universitari Parc Tauli	Barcelona, Spain	Research Coordinator	Aqueous-PREP
Yeray	Maldonado-Sotoca			Hospital Universitari Parc Tauli	Barcelona, Spain	Research Coordinator	Aqueous-PREP
Alfons	Gasset-Teixidor			Hospital Universitari Parc Tauli	Barcelona, Spain	Research Coordinator	Aqueous-PREP
Jorge	Blasco-Moreu			Hospital Universitari Parc Tauli	Barcelona, Spain	Research Coordinator	Aqueous-PREP
Núria	Fernández-Poch			Hospital Universitari Parc Tauli	Barcelona, Spain	Research Coordinator	Aqueous-PREP
Josep	Rodoreda-Puigdemasa			Hospital Universitari Parc Tauli	Barcelona, Spain	Research Coordinator	Aqueous-PREP
Arnau	Verdaguer-Figuerola			Hospital Universitari Parc Tauli	Barcelona, Spain	Research Coordinator	Aqueous-PREP
Heber	Enrique Cueva-Sevieri			Hospital Universitari Parc Tauli	Barcelona, Spain	Research Coordinator	Aqueous-PREP
Santiago	Garcia-Gimenez			Hospital Universitari Parc Tauli	Barcelona, Spain	Research Coordinator	Aqueous-PREP
Ernesto	Guerra-Farfan			Vall d'Hebron University Hospital	Barcelona, Spain	Local Principal Investigator	Aqueous-PREP
Jordi	Tomas-Hernandez			Vall d'Hebron University Hospital	Barcelona, Spain	Research Coordinator	Aqueous-PREP
Jordi	Teixidor-Serra			Vall d'Hebron University Hospital	Barcelona, Spain	Research Coordinator	Aqueous-PREP

*Indicates required information. Only first name, last name, and suffix will appear in PubMed.

***First Name and Middle Initial(s)**	***Last Name**	***Suffix (eg, Jr, III)**	Academic Degrees	Institution	Location (city, state/province, country)	Role or Contribution, eg, chair, principal investigator	Group (if more than 1 Group listed in the byline) and/or Subgroup (eg, Steering Committee)
Vicente	Molero-Garcia			Vall d'Hebron University Hospital	Barcelona, Spain	Research Coordinator	Aqueous-PREP
Jordi	Selga-Marsa			Vall d'Hebron University Hospital	Barcelona, Spain	Research Coordinator	Aqueous-PREP
Juan	Antonio Porcel-Vasquez			Vall d'Hebron University Hospital	Barcelona, Spain	Research Coordinator	Aqueous-PREP
Jose	Vicente Andres-Peiro			Vall d'Hebron University Hospital	Barcelona, Spain	Research Coordinator	Aqueous-PREP
Joan	Minguell-Monyart			Vall d'Hebron University Hospital	Barcelona, Spain	Research Coordinator	Aqueous-PREP
Jorge	Nuñez-Camarena			Vall d'Hebron University Hospital	Barcelona, Spain	Research Coordinator	Aqueous-PREP
Maria	del Mar Villar-Casares			Vall d'Hebron University Hospital	Barcelona, Spain	Research Coordinator	Aqueous-PREP
Jaume	Mestre-Torres			Vall d'Hebron University Hospital	Barcelona, Spain	Research Coordinator	Aqueous-PREP
Pilar	Lalueza-Broto			Vall d'Hebron University Hospital	Barcelona, Spain	Research Coordinator	Aqueous-PREP
Felipe	Moreira-Borim			Vall d'Hebron University Hospital	Barcelona, Spain	Research Coordinator	Aqueous-PREP
Yaiza	Garcia-Sanchez			Vall d'Hebron University Hospital	Barcelona, Spain	Research Coordinator	Aqueous-PREP
Nicholas M.	Romeo			MetroHealth Medical Center	Cleveland, OH	Local Principal Investigator	PREPARE
Heather A.	Vallier			MetroHealth Medical Center	Cleveland, OH	Co-Investigator	PREPARE
Mary A.	Breslin			MetroHealth Medical Center	Cleveland, OH	Research Coordinator	PREPARE
Joanne	Fraifogl			MetroHealth Medical Center	Cleveland, OH	Research Assistant	PREPARE
Eleanor S.	Wilson			MetroHealth Medical Center	Cleveland, OH	Research Assistant	PREPARE
Leanne K.	Wadenpfuhl			MetroHealth Medical Center	Cleveland, OH	Research Assistant	PREPARE
Paul G.	Halliday			MetroHealth Medical Center	Cleveland, OH	Research Assistant	PREPARE
Isabella	Heimke			MetroHealth Medical Center	Cleveland, OH	Research Assistant	PREPARE
Darius G.	Viskontas			FRASER HEALTH AUTHORITY/Royal Columbian Hospital	New Westminster, BC	Local Principal Investigator	PREPARE
Kelly L.	Apostle			FRASER HEALTH AUTHORITY/Royal Columbian Hospital	New Westminster, BC	Co-Investigator	PREPARE

*Indicates required information. Only first name, last name, and suffix will appear in PubMed.

*First Name and Middle Initial(s)	*Last Name	*Suffix (eg, Jr, III)	Academic Degrees	Institution	Location (city, state/province, country)	Role or Contribution, eg, chair, principal investigator	Group (if more than 1 Group listed in the byline) and/or Subgroup (eg, Steering Committee)
Dory S.	Boyer			FRASER HEALTH AUTHORITY/Royal Columbian Hospital	New Westminster, BC	Co-Investigator	PREPARE
Farhas O.	Moola			FRASER HEALTH AUTHORITY/Royal Columbian Hospital	New Westminster, BC	Co-Investigator	PREPARE
Bertrand H.	Perey			FRASER HEALTH AUTHORITY/Royal Columbian Hospital	New Westminster, BC	Co-Investigator	PREPARE
Trevor B.	Stone			FRASER HEALTH AUTHORITY/Royal Columbian Hospital	New Westminster, BC	Co-Investigator	PREPARE
H. Michael	Lemke			FRASER HEALTH AUTHORITY/Royal Columbian Hospital	New Westminster, BC	Co-Investigator	PREPARE
Mauri	Zomar			FRASER HEALTH AUTHORITY/Royal Columbian Hospital	New Westminster, BC	Research Coordinator	PREPARE
Ella	Spicer			FRASER HEALTH AUTHORITY/Royal Columbian Hospital	New Westminster, BC	Research Coordinator	PREPARE
Chen "Brenda"	Fan			FRASER HEALTH AUTHORITY/Royal Columbian Hospital	New Westminster, BC	Research Assistant	PREPARE
Krysten	Payne			FRASER HEALTH AUTHORITY/Royal Columbian Hospital	New Westminster, BC	Research Assistant	PREPARE
Kevin	Phelps			Carolinas Medical Center, Atrium Health Musculoskeletal Institute	Charlotte, NC	Local Principal Investigator	PREPARE

Supplemental Online Content: Nonauthor Collaborators

*Indicates required information. Only first name, last name, and suffix will appear in PubMed.

*First Name and Middle Initial(s)	*Last Name	*Suffix (eg, Jr, III)	Academic Degrees	Institution	Location (city, state/province, country)	Role or Contribution, eg, chair, principal investigator	Group (if more than 1 Group listed in the byline) and/or Subgroup (eg, Steering Committee)
Michael	Bosse			Carolinas Medical Center, Atrium Health Musculoskeletal Institute	Charlotte, NC	Co-Investigator	PREPARE
Madhav	Karunakar			Carolinas Medical Center, Atrium Health Musculoskeletal Institute	Charlotte, NC	Co-Investigator	PREPARE
Laurence	Kempton			Carolinas Medical Center, Atrium Health Musculoskeletal Institute	Charlotte, NC	Co-Investigator	PREPARE
Stephen	Sims			Carolinas Medical Center, Atrium Health Musculoskeletal Institute	Charlotte, NC	Co-Investigator	PREPARE
Joseph	Hsu			Carolinas Medical Center, Atrium Health Musculoskeletal Institute	Charlotte, NC	Co-Investigator	PREPARE
Rachel	Seymour			Carolinas Medical Center, Atrium Health Musculoskeletal Institute	Charlotte, NC	Manager	PREPARE
Christine	Churchill			Carolinas Medical Center, Atrium Health Musculoskeletal Institute	Charlotte, NC	Research Coordinator	PREPARE
Claire	Bartel			Carolinas Medical Center, Atrium Health Musculoskeletal Institute	Charlotte, NC	Research Assistant	PREPARE
Robert Miles	Mayberry			Carolinas Medical Center, Atrium Health Musculoskeletal Institute	Charlotte, NC	Research Assistant	PREPARE
Maggie	Brownrigg			Carolinas Medical Center, Atrium Health Musculoskeletal Institute	Charlotte, NC	Research Assistant	PREPARE

*Indicates required information. Only first name, last name, and suffix will appear in PubMed.

*First Name and Middle Initial(s)	*Last Name	*Suffix (eg, Jr, III)	Academic Degrees	Institution	Location (city, state/province, country)	Role or Contribution, eg, chair, principal investigator	Group (if more than 1 Group listed in the byline) and/or Subgroup (eg, Steering Committee)
Cara	Girardi			Carolinas Medical Center, Atrium Health Musculoskeletal Institute	Charlotte, NC	Research Assistant	PREPARE
Ada	Mayfield			Carolinas Medical Center, Atrium Health Musculoskeletal Institute	Charlotte, NC	Research Assistant	PREPARE
Juliette	Sweeney			Carolinas Medical Center, Atrium Health Musculoskeletal Institute	Charlotte, NC	Research Assistant	PREPARE
Hannah	Pollock			Carolinas Medical Center, Atrium Health Musculoskeletal Institute	Charlotte, NC	Research Assistant	PREPARE
Robert A.	Hymes			Inova Fairfax Medical Campus	Falls Church, VA	Local Principal Investigator	PREPARE
Cary C.	Schwartzbach			Inova Fairfax Medical Campus	Falls Church, VA	Co-Investigator	PREPARE
Jeff E.	Schulman			Inova Fairfax Medical Campus	Falls Church, VA	Co-Investigator	PREPARE
A. Stephen	Malekzadeh			Inova Fairfax Medical Campus	Falls Church, VA	Co-Investigator	PREPARE
Michael A.	Holzman			Inova Fairfax Medical Campus	Falls Church, VA	Co-Investigator	PREPARE
Greg E.	Gaski			Inova Fairfax Medical Campus	Falls Church, VA	Co-Investigator	PREPARE
Jonathan	Wills			Inova Fairfax Medical Campus	Falls Church, VA	Co-Investigator	PREPARE
Lolita	Ramsey			Inova Fairfax Medical Campus	Falls Church, VA	Manager	PREPARE
James S.	Ahn			Inova Fairfax Medical Campus	Falls Church, VA	Research Coordinator	PREPARE
Farhanaz	Panjshiri			Inova Fairfax Medical Campus	Falls Church, VA	Research Coordinator	PREPARE
Sharmistha	Das			Inova Fairfax Medical Campus	Falls Church, VA	Research Coordinator	PREPARE
Antoinisha D.	English			Inova Fairfax Medical Campus	Falls Church, VA	Research Coordinator	PREPARE
Sharon M.	Haaser			Inova Fairfax Medical Campus	Falls Church, VA	Research Assistant	PREPARE
Jaslynn A. N.	Cuff			Inova Fairfax Medical Campus	Falls Church, VA	Research Assistant	PREPARE
Holly	Pilson			Wake Forest Baptist Health	Winston-Salem, NC	Local Principal Investigator	PREPARE
Eben A.	Carroll			Wake Forest Baptist Health	Winston-Salem, NC	Co-Investigator	PREPARE
Jason J.	Halvorson			Wake Forest Baptist Health	Winston-Salem, NC	Co-Investigator	PREPARE
Sharon	Babcock			Wake Forest Baptist Health	Winston-Salem, NC	Co-Investigator	PREPARE

Supplemental Online Content: Nonauthor Collaborators

*Indicates required information. Only first name, last name, and suffix will appear in PubMed.

*First Name and Middle Initial(s)	*Last Name	*Suffix (eg, Jr, III)	Academic Degrees	Institution	Location (city, state/province, country)	Role or Contribution, eg, chair, principal investigator	Group (if more than 1 Group listed in the byline) and/or Subgroup (eg, Steering Committee)
J. Brett	Goodman			Wake Forest Baptist Health	Winston-Salem, NC	Research Coordinator	PREPARE
Martha B.	Holden			Wake Forest Baptist Health	Winston-Salem, NC	Research Assistant	PREPARE
Debra	Bullard			Wake Forest Baptist Health	Winston-Salem, NC	Research Assistant	PREPARE
Wendy	Williams			Wake Forest Baptist Health	Winston-Salem, NC	Research Assistant	PREPARE
Taylor	Hill			Wake Forest Baptist Health	Winston-Salem, NC	Research Assistant	PREPARE
Ariel	Brotherton			Wake Forest Baptist Health	Winston-Salem, NC	Research Assistant	PREPARE
Thomas F.	Higgins			University of Utah	Salt Lake City, Utah	Local Principal Investigator	PREPARE
Justin M.	Haller			University of Utah	Salt Lake City, Utah	Co-Investigator	PREPARE
David L.	Rothberg			University of Utah	Salt Lake City, Utah	Co-Investigator	PREPARE
Lucas S.	Marchand			University of Utah	Salt Lake City, Utah	Co-Investigator	PREPARE
Ashley	Neese			University of Utah	Salt Lake City, Utah	Manager	PREPARE
Mark	Russell			University of Utah	Salt Lake City, Utah	Research Coordinator	PREPARE
Zachary M	Olsen			University of Utah	Salt Lake City, Utah	Research Coordinator	PREPARE
Abby V.	McGowan			University of Utah	Salt Lake City, Utah	Research Coordinator	PREPARE
Sophia	Hill			University of Utah	Salt Lake City, Utah	Research Coordinator	PREPARE
I. Leah	Gitajn			Dartmouth-Hitchcock Medical Center	Lebanon, NH	Local Principal Investigator	PREPARE
Marcus	Coe			Dartmouth-Hitchcock Medical Center	Lebanon, NH	Co-Investigator	PREPARE
Kevin	Dwyer			Dartmouth-Hitchcock Medical Center	Lebanon, NH	Co-Investigator	PREPARE
Devin	Mullin			Dartmouth-Hitchcock Medical Center	Lebanon, NH	Research Coordinator	PREPARE
Clifford A.	Reilly			Dartmouth-Hitchcock Medical Center	Lebanon, NH	Research Assistant	PREPARE
Peter	DePalo			Dartmouth-Hitchcock Medical Center	Lebanon, NH	Research Assistant	PREPARE
Amy E.	Hall			Dartmouth-Hitchcock Medical Center	Lebanon, NH	Research Assistant	PREPARE
Rebecca E.	Dabrowski			Dartmouth-Hitchcock Medical Center	Lebanon, NH	Research Assistant	PREPARE

Supplemental Online Content: Nonauthor Collaborators

*Indicates required information. Only first name, last name, and suffix will appear in PubMed.

***First Name and Middle Initial(s)**	***Last Name**	***Suffix (eg, Jr, III)**	Academic Degrees	Institution	Location (city, state/province, country)	Role or Contribution, eg, chair, principal investigator	Group (if more than 1 Group listed in the byline) and/or Subgroup (eg, Steering Committee)
Theresa A.	Chockbengboun			Dartmouth-Hitchcock Medical Center	Lebanon, NH	Research Assistant	PREPARE
Marilyn	Heng			Massachusetts General Hospital	Boston, MA	Local Principal Investigator	PREPARE
Mitchel B.	Harris			Massachusetts General Hospital	Boston, MA	Co-Investigator	PREPARE
R. Malcolm	Smith			Massachusetts General Hospital	Boston, MA	Co-Investigator	PREPARE
David W.	Lhowe			Massachusetts General Hospital	Boston, MA	Co-Investigator	PREPARE
John G.	Esposito			Massachusetts General Hospital	Boston, MA	Co-Investigator	PREPARE
Mira	Bansal			Massachusetts General Hospital	Boston, MA	Research Assistant	PREPARE
Michael	McTague			Massachusetts General Hospital	Boston, MA	Research Director	PREPARE
Ahmad	Alnasser			Massachusetts General Hospital	Boston, MA	Research Assistant	PREPARE
Patrick F.	Bergin			University of Mississippi Medical Center	Jackson, MS	Local Principal Investigator	PREPARE
George V.	Russell			University of Mississippi Medical Center	Jackson, MS	Co-Investigator	PREPARE
Matthew L.	Graves			University of Mississippi Medical Center	Jackson, MS	Co-Investigator	PREPARE
John	Morellato			University of Mississippi Medical Center	Jackson, MS	Co-Investigator	PREPARE
Heather K.	Champion			University of Mississippi Medical Center	Jackson, MS	Research Assistant	PREPARE
Leslie N.	Johnson			University of Mississippi Medical Center	Jackson, MS	Research Coordinator	PREPARE
Sheketha L.	McGee			University of Mississippi Medical Center	Jackson, MS	Manager	PREPARE
Eldrin L.	Bhanat			University of Mississippi Medical Center	Jackson, MS	Research Coordinator	PREPARE
Josny	Thimothee			University of Mississippi Medical Center	Jackson, MS	Research Coordinator	PREPARE
Jackie	Serrano			University of Mississippi Medical Center	Jackson, MS	Research Coordinator	PREPARE

Supplemental Online Content: Nonauthor Collaborators

*Indicates required information. Only first name, last name, and suffix will appear in PubMed.

*First Name and Middle Initial(s)	*Last Name	*Suffix (eg, Jr, III)	Academic Degrees	Institution	Location (city, state/province, country)	Role or Contribution, eg, chair, principal investigator	Group (if more than 1 Group listed in the byline) and/or Subgroup (eg, Steering Committee)
Samir	Mehta			University of Pennsylvania	Philadelphia, PA	Local Principal Investigator	PREPARE
Derek	Donehan			University of Pennsylvania	Philadelphia, PA	Co-Investigator	PREPARE
Jaimo	Ahn			University of Pennsylvania	Philadelphia, PA	Co-Investigator	PREPARE
Annamarie	Horan			University of Pennsylvania	Philadelphia, PA	Manager	PREPARE
Mary	Dooley			University of Pennsylvania	Philadelphia, PA	Research Assistant	PREPARE
Ashley	Kuczinski			University of Pennsylvania	Philadelphia, PA	Research Coordinator	PREPARE
Ashley	Iwu			University of Pennsylvania	Philadelphia, PA	Research Coordinator	PREPARE
David	Potter			Sanford Health	Sioux Falls, SD	Local Principal Investigator	PREPARE
Robert	VanDemark	III		Sanford Health	Sioux Falls, SD	Co-Investigator	PREPARE
Branden	Pfaff			Sanford Health	Sioux Falls, SD	Research Coordinator	PREPARE
Troy	Hollinsworth			Sanford Health	Sioux Falls, SD	Research Coordinator	PREPARE
Kristi	Atkins			Sanford Health	Sioux Falls, SD	Research Coordinator	PREPARE
Michael J.	Weaver			Brigham Women's Hospital	Boston, MA	Local Principal Investigator	PREPARE
Arvind G.	von Keudell			Brigham Women's Hospital	Boston, MA	Co-Investigator	PREPARE
Elizabeth M.	Allen			Brigham Women's Hospital	Boston, MA	Research Assistant	PREPARE
Abigail E.	Sagona			Brigham Women's Hospital	Boston, MA	Research Assistant	PREPARE
Todd	Jaeblon			University of Maryland Prince George’s Capital Region Health	Cheverly, MD	Local Principal Investigator	PREPARE
Robert	Beer			University of Maryland Prince George’s Capital Region Health	Cheverly, MD	Co-Investigator	PREPARE
Haley K.	Demyanovich			University of Maryland Prince George’s Capital Region Health	Cheverly, MD	Research Coordinator	PREPARE
Brent	Bauer			University of Maryland Prince George’s Capital Region Health	Cheverly, MD	Research Coordinator	PREPARE
Sean	Meredith			University of Maryland Prince George’s Capital Region Health	Cheverly, MD	Research Coordinator	PREPARE
Autumn	Stone			University of Maryland Prince George’s Capital Region Health	Cheverly, MD	Research Coordinator	PREPARE

Supplemental Online Content: Nonauthor Collaborators

*Indicates required information. Only first name, last name, and suffix will appear in PubMed.

***First Name and Middle Initial(s)**	***Last Name**	***Suffix (eg, Jr, III)**	Academic Degrees	Institution	Location (city, state/province, country)	Role or Contribution, eg, chair, principal investigator	Group (if more than 1 Group listed in the byline) and/or Subgroup (eg, Steering Committee)
Mark J.	Gage			Duke University Hospital	Durham, NC	Local Principal Investigator	PREPARE
Rachel M.	Reilly			Duke University Hospital	Durham, NC	Co-Investigator	PREPARE
Cindy	Sparrow			Duke University Hospital	Durham, NC	Research Coordinator	PREPARE
Ariana	Paniagua			Duke University Hospital	Durham, NC	Research Coordinator	PREPARE
